# Localized Tissue-Specific Gene Expression and Gene Duplications are Important Sources of Social Morph Differences in a Social Bumblebee

**DOI:** 10.1093/molbev/msaf063

**Published:** 2025-03-27

**Authors:** Hongfei Xu, Thomas J Colgan

**Affiliations:** Institute of Organismic and Molecular Evolution, Johannes Gutenberg University Mainz, 55128 Mainz, Germany; Institute of Organismic and Molecular Evolution, Johannes Gutenberg University Mainz, 55128 Mainz, Germany; Institute for Quantitative and Computational Biosciences (IQCB), Johannes Gutenberg University Mainz, 55128 Mainz, Germany

**Keywords:** phenotypic polymorphism, bumblebees, transcriptomics, gene duplication, tissue-specificity

## Abstract

Understanding the expression of multiple behaviorally and morphologically distinct phenotypes from a single genome represents a fundamental topic in evolutionary biology. Central to the complication of expressing phenotypes, which may differ in their optima, is the sharing of largely the same genome, which is predicted to manifest in conflict at the genomic level. This is particularly true for social insects where molecular mechanisms, such as differential gene expression, contribute to observed phenotypic differences between reproductive and nonreproductive morphs. In comparison, other mechanisms, such as tissue-specific expression and gene duplications, have been posited as contributing to social morph differences yet formal investigations are limited. Here, using a combination of transcriptomics for multiple tissues and comparative genomics, we show that in a social bumblebee, the strongest differences in gene expression are found in reproductive tissues, such as the spermatheca, an organ previously believed as vestigial in workers but recently shown as functional. In comparison, we find modest expression differences in genes between queens and workers for the brain, fat body, and ovary, which are traditionally investigated in social evolution. Interestingly, morph-biased genes in these three tissues display higher tissue-specificity suggesting that while social morphs may express a shared core transcriptome, localized expression profiles may contribute to phenotypic differences. We also find evidence of differential usage of duplicated genes by queens and workers, highlighting structural variants as a contributing factor to morph differences. Collectively, our findings highlight how social insects can utilize tissue-specific gene regulation and structural variants to contribute to phenotypic differences.

## Introduction

Within a species, different morphs, such as sexes, may express different phenotypic traits, which allow them to occupy or perform specialized functional roles (West-Eberhard 2003). Due to intraspecific variation in functional roles, morphs may express distinct role-associated phenotypic traits, which may manifest as morphological, physiological, or behavioral differences. As a consequence, morphs sharing the same genome may experience different selective pressures, inhibiting each from reaching their phenotypic optima, and resulting in potential intralocus conflict at the genomic level. As such conflict may contribute to differential effects on morph viability and survival, mechanisms have evolved to resolve conflict (Pennell et al. 2018). The most prominent and well-studied means of resolution is differential gene expression, which is posited to lead to the evolution of dimorphic phenotypic traits (Orr and Goodisman 2023). However, conflict may also be resolved through structural variants, such as the evolution of morph-specific genomic architecture, such as sex chromosomes, or through gene duplication events (Bonduriansky and Chenoweth 2009), whereby the duplication of a previously antagonistic allele relaxes selection acting on one copy allowing for structural and potential functional diversification, which can contribute to differences observed at the phenotypic level.

Exemplar systems for studying the contribution of genome expression and structural variation underlying complex phenotypic traits are social insects, which include members of the social Hymenoptera (ants, some bees, and wasps) and Isoptera (termites). In eusocial members of the Hymenoptera, a reproductive division of labor has evolved within the female sex where behaviorally and morphologically distinct social morphs (traditionally referred to as “castes”) undertake complementary tasks that contribute to colony fitness (Wilson 1971). Within these colonies, the influence of certain social conditions can lead to social morphs having different functional fates, despite sharing mostly the same genetic background. For example, longer lived morphs, known as queens, are fertile and primarily responsible for the main reproductive output of the colony, while shorter lived workers mainly perform altruistic tasks, such as foraging, nursing, and nest defense (Wilson 1971). Since individuals of distinct social insect morphs share largely the same genome, phenotypic differences are mainly derived from differential gene expression (Corona et al. 2016). Given the key role of gene regulation in the expression of queen and worker morphs, a large body of research has been performed analyzing and comparing the transcriptomes of queens and workers, identifying an extensive compendium of genes differentially expressed between social morphs in bees (Harrison et al. 2015; Warner et al. 2019; Zhang et al. 2022), ants (Warner et al. 2019; Qiu et al. 2022), and wasps (Taylor et al. 2021), which are involved in a range of biological processes, including reproduction, metabolism, chemical communication, and immunity (Orr and Goodisman 2023). The molecular bases of complex phenotypic differences between social morphs may, therefore, differ in spatial scales across multiple tissues. Consequently, studies limited to a single tissue or using the whole individual may underestimate or lead to the omission of certain genes that are important for morph differences (Johnson et al. 2013). Furthermore, for many species, gene expression differences between morphs can be confounded by differences in the age, reproductive and insemination status, as well as anatomical differences in shared traits (Hoffman and Goodisman 2007; Harrison et al. 2015). These aspects are particularly difficult to control when comparing queens and workers as: (i) queens and workers, generally, differ extensively in lifespan with queens having extended longevity compared to short-lived workers; (ii) the window of sexual production may be very small limiting access to new queen sampling meaning comparisons are performed on morphs sampled across generations (i.e. intergenerational comparisons between mothers and daughters); and (iii) for many social insects, workers are generally functionally sterile, in some cases lacking reproductive organs, and do not mate. Indeed, life-time unmatedness along with pre-imaginal morph determination, have been proposed as criteria for the classification of a social insect species as a superorganism (Boomsma and Gawne 2018), a higher level of social complexity. Therefore, ultimately, our understanding of intrinsic differences between queen and worker phenotypes may be underestimated due to restricted profiling of specific tissues linked to morph-related traits, or potentially confounded as a consequence of comparing morphs that differ with respect to anatomy, age and/or reproductive status.

Within social insects, bumblebees (*Bombus* species) represent an increasingly powerful model for examining the molecular-genetic processes associated with social morph differences. Bumblebees are a taxon-rich genus, consisting of ∼300 described species (Williams 1998, 2023) and are important ecological pollinators that inhabit a diverse range of habitats (Alford 1975; Goulson 2003). All extant bumblebee species derive from a social common ancestor with eusociality secondarily lost in certain species (Almeida et al. 2023). For social bumblebees, comparisons between social morphs benefit from: first, phenotypic differences in terms of morphology are less pronounced as queens and workers are anatomically similar with most differences in morphology due to relative size differences (del Castillo and Fairbairn 2012); second, the relative small colony size, annual life-cycle, and ability to keep colonies in a laboratory setting means it is also feasible to sample age-controlled queens and workers; third, like queens, workers retain a smaller in size but functional spermatheca (Schoeters and Billen 2000; Zhuang et al. 2023), an organ lost or degenerated in workers of more advanced insect societies (Snodgrass 1956; Gobin et al. 2006), with both bumblebee queens and workers possessing the capacity to mate and found colonies (Zhuang et al. 2023); and fourth, studies on the molecular biology and genetics of bumblebees have greatly benefitted from the generation of genomic resources, including chromosomal-level reference genome assemblies and high-quality gene model predictions (Sadd et al. 2015), including assemblies for representatives from each subgenus (Sun et al. 2021), providing the platform to examine and identify structural variants, such as duplications, which may display morph-biased expression profiles. Transcriptomic-based analyses have previously identified genes associated with phenotypic polymorphism and morph differences but suffered from low sample sizes or from comparisons of whole-bodied individuals, losing tissue-specific signals as a consequence (Colgan et al. 2011; Harrison et al. 2015). In addition, the role, if any, that gene duplications contribute to morph differences in the social Hymenoptera is largely understudied (but see Chau and Goodisman 2017).

To address this gap in our knowledge, we investigated morph-based differences in *Bombus terrestris*, using a publicly available transcriptomic dataset (*n* = 256 RNA-seq libraries; Zhuang et al. 2023) comprising of four tissues (brains, fat bodies, ovaries, and other reproductive tissues (RTs), including the spermatheca) from queens and workers. Our dataset consisted of queens and workers of similar age collected across 3 individual time-points of ovarian development (stage I: days 3 to 4 post-eclosion; stage II: days 4 to 5 post-eclosion; and stage IV: days 7 post-eclosion), which allowed us to control for the influence of differences in ovarian development in our analysis. In addition, the dataset also included both queens and workers that were artificially inseminated and noninseminated, respectively, which allowed for accounting for potential differences in gene expression associated with insemination status. Accounting for these factors in our analyses allows us to avoid conflating morph differences with differences in gene expression explained by variation in ovarian development or insemination status, while also allowing us to maximize our sample size, increasing our ability to accurately detect and quantify intrinsic differences between social morphs. Using all 256 samples of four tissues, we first quantified differentially expressed genes (DEGs) between queens and workers in each tissue, allowing us to understand spatial differences in morph-biased expression profiles. Second, we further examined morph-biased genes for tissue-specificity, to understand if differences between morphs were found globally conserved across tissues or localized in terms of expression, which may contribute to conflict resolution. Lastly, as gene duplications may serve as an additional mechanism that contributes to phenotypic differences between queens and workers, we assessed the presence of *Bombus*-specific duplicated genes and determined whether such genes displayed patterns of divergent expression profiles between queens and workers, which may indicate patterns of subfunctionalization or neofunctionalization, and contribute to the expression of diverse social phenotypes (Birchler and Yang 2022).

## Results

### Largest Morph-Biased Gene Expression Detected in Spermatheca and Associated Reproductive Tissues

**Fig. 1.**
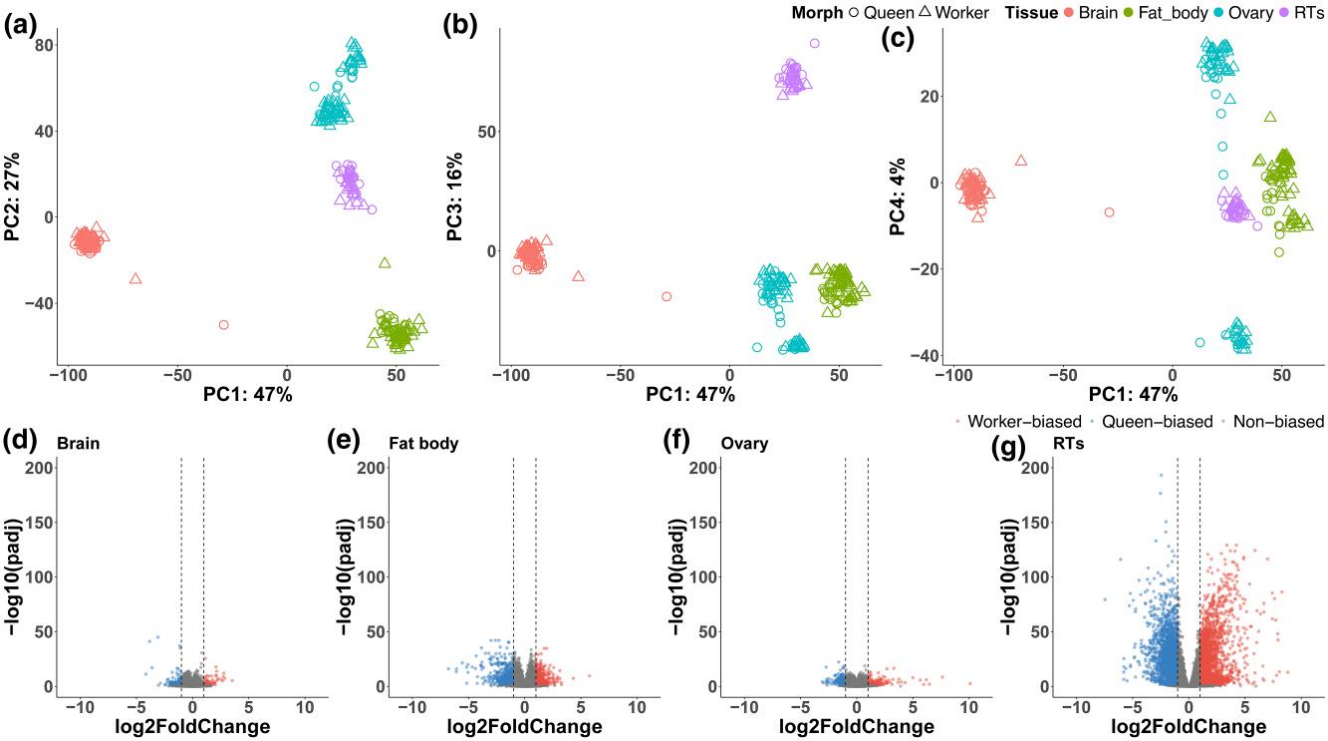
Spermatheca and associated tissues show the largest differences in morph-biased gene expression across bumblebee tissues. a-c) Scatterplots displaying the first 4 principal components calculated from a PCA performed on normalized gene-level counts, which collectively explain 94% of the variance, and show that individual samples clustered based on tissue of origin. For each plot, the first PC, as well as the proportion of variance it explains, are provided on the *x* axis while either the second, third, or fourth PC are provided on the *y* axis, respectively. Each dot represents an individual sample while social morph is indicated by a distinct shape and the tissue of origin is indicated by a distinct color. d-g) Volcano plots displaying the number of DEGs (Benjamini–Hochberg adjusted *P* (padj) < 0.05, |log2FoldChange| ≥ 1), as well as their direction of expression, between bumblebee workers and queens for each of 4 tissues (brain, fat body, ovary, and RTs). In each plot, worker-, queen-, and non-biased genes are represented by dots with distinct color. Black dashed vertical lines indicate log2FoldChange thresholds for worker-biased (log2FoldChange = 1) and queen-biased (log2FoldChange = −1) gene expression. For each gene, log2FoldChange is shown on the *x* axis with -log10-transformed padj values provided on the *y* axis.

To identify gene expression differences between queens and workers of the large earth or buff-tailed bumblebee, *B. terrestris*, we analyzed transcriptomic data collected from queens and workers from four tissues: brain, fat body, ovary, and other reproductive tissues (RTs, including a pool of the spermatheca, vagina, and median oviduct). The dataset was generated as part of a previous study (Zhuang et al. 2023), which examined transcriptional changes in similar-aged queens and workers in response to insemination. Here, we are using it to understand intrinsic differences between queens and workers when differences in age and reproductive status are accounted for. To determine if samples clustered together based on tissue, we first performed a principal component analysis (PCA) on normalized (variance stabilized transformed) gene-level counts finding that the first principal component (PC1; Fig. 1a to c; supplementary table S1, Supplementary Material online), which explained 47% of the variance, separated brains from the other three tissues. In comparison, the ovaries and fat bodies were broadly separated from the other two tissues by PC2 (variance explained = 27%; Fig. 1a). Given the intrinsic transcriptional differences between tissues, we performed independent differential expression analyses for each. We identified considerable variation in the number of DEGs within tissues between bumblebee morphs (Fig. 1d to g). The largest transcriptional differences between queens and workers were identified in the RTs with a total of 4,169 significantly DEGs (LRT: Benjamini–Hochberg adjusted *P*-value (padj) < 0.05, absolute log2FoldChange ≥ 1). Within this number, we identified a similar number of worker- (*n* = 2,170) and queen-biased genes (*n* = 1,999; Fig. 1g; supplementary table S2, Supplementary Material online). In comparison, we identified 4.7 to 20 times fewer morph-biased genes in the other three tissues examined with: 215 DEGs detected in the brain (worker-biased = 56; queen-biased = 159); 887 detected in the fat body (worker-biased = 392; queen-biased = 495); and 320 detected in the ovary (worker-biased = 133; queen-biased = 187; Fig. 1d to f;  supplementary table S2, Supplementary Material online).

### Morph-Biased Genes Have Higher Tissue-specific Expression

**Fig. 2.**
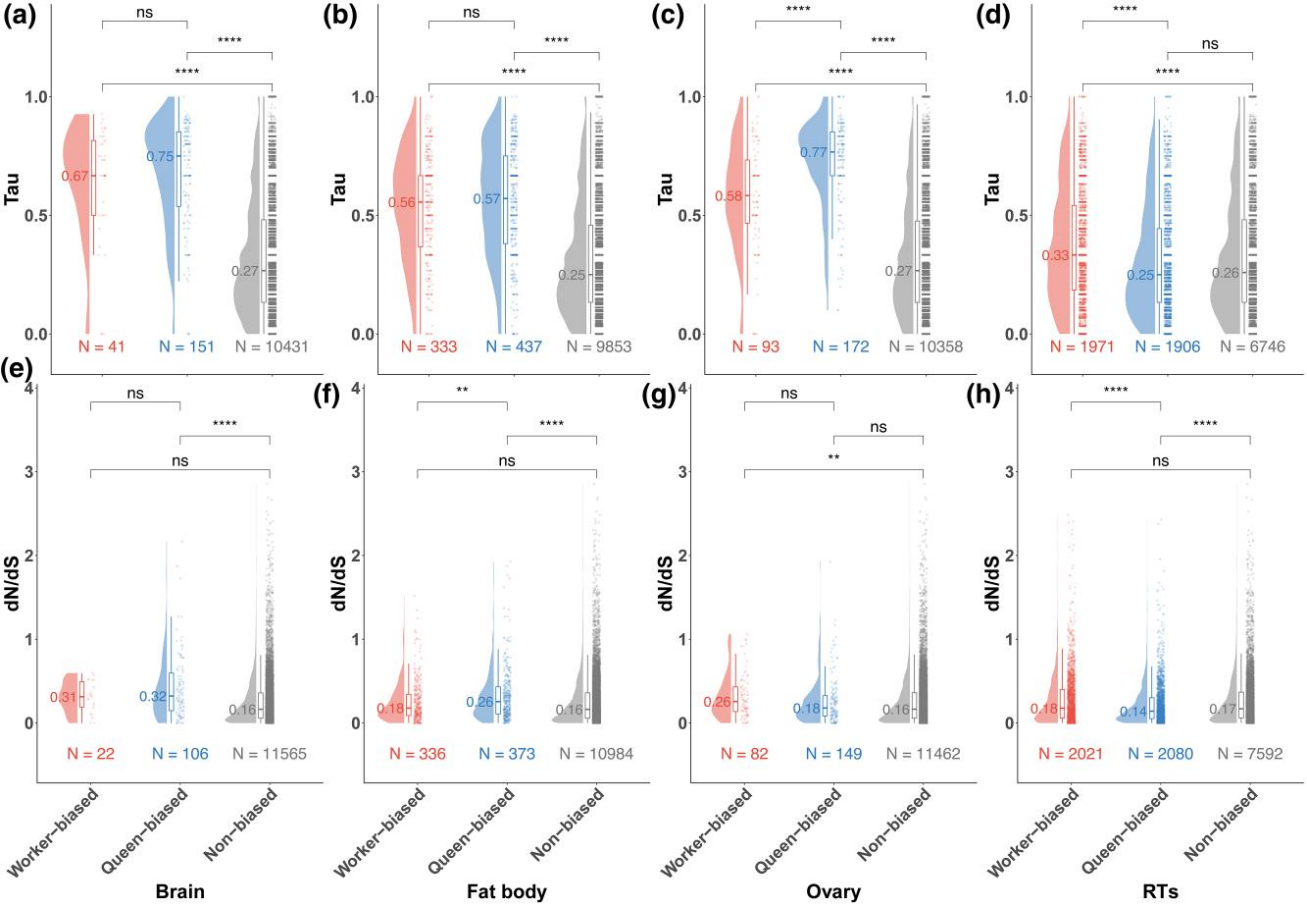
Morph-biased genes generally show greater tissue-specificity but differ in levels of evolutionary conservation. a-d) Raincloud plots display the distribution of tissue-specificity (tau value, *y* axis) for worker-, queen-, and non-biased genes for each of 4 tissues (brain, fat body, ovary, and RTs). The “cloud” part represents the kernel density estimation of the data distribution, the “rain” part consists of individual data points (jittered for visibility). The median tau value for each gene category is represented as a single line inside each box part with the actual median displayed on the left side of this line while the number of genes per gene category are shown below each corresponding plot; and (e-h) Raincloud plots display the estimates of dN/dS ratios generated from comparing CDS between *B. terrestris* and the closely related *B. impatiens*, for worker-, queen-, and non-biased genes for each tissue (brain, fat body, ovary, and RTs). The “cloud” part represents the kernel density estimation of the data distribution, the “rain” part consists of individual data points (jittered for visibility). The median dN/dS value for each set is represented as a single line inside each box part with the actual median displayed on the left side of this line while the number of genes per set shown below the corresponding plot. Tests of significance for differences in tissue-specificity and divergence between gene categories were performed using Wilcoxon rank-sum tests, the results, of which for pairwise comparisons with Bonferroni correction between gene categories are shown (ns = not significant, * = Bonferroni-adjusted *P* < 0.05, ** = Bonferroni-adjusted *P* < 0.01, *** = Bonferroni-adjusted *P* < 0.001, and **** = Bonferroni-adjusted *P* < 0.0001).

Given that queens and workers share the same genome, it may be expected that they largely express a conserved set of genes (“core” transcriptome) with morph-biased genes displaying more localized or tissue-specific expression (Warner et al. 2019; Witt et al. 2021). To test this, we calculated and compared tau values, which provide a measure of tissue-specificity, between worker-biased genes, queen-biased genes, and non-biased genes (supplementary table S3, Supplementary Material online). We found that, on average, queen- and worker-biased genes had significantly higher tau values reflecting higher tissue-specificity than non-biased genes, which was a conserved pattern found across tissues with the exception of queen-biased genes in the RTs (Wilcoxon rank-sum test, Bonferroni-adjusted *P* < 1e-11; Fig. 2a to d).

As tissue-specific expression may be associated with gene duplications (De Smet et al. 2013; Mantica et al. 2024), we investigated if the higher tissue-specificity identified in morph-biased genes was a potential consequence of morph-biased genes consisting of more paralogues. We, therefore, compared the proportion of multicopy (paralogues) and single-copy (orthologues) genes, as identified within a representative number of hymenopteran species (Materials and Methods: Identification of lineage-specific duplicated genes), within morph-biased genes for each tissue. We found that morph-biased genes contained a significantly higher proportion of multicopy genes than expected (χ^2^ test, *P* < 1e-8) in the brain (expected proportion of paralogues = 0.75; observed proportion of paralogues = 0.95), fat body (expected = 0.75; observed = 0.88), and ovary (expected = 0.75; observed = 0.89). We found a similar pattern for worker-biased genes in RTs with higher proportions of multicopy genes, albeit at a lower scale (expected = 0.74; observed = 0.78). The opposite was true for queen-biased genes, with significantly lower than expected proportions of multicopy genes being identified (expected = 0.74; observed = 0.72). Across all tissues, both queens and workers, with the exception of queen-biased genes in the RTs, appear to preferentially express more paralogues.

### Queen-Biased Genes Display Stronger Selective Constraints in RTs

As morph-biased genes in social insects have been shown to have signatures consistent with faster evolution (Hunt et al. 2011, 2013; Warner et al. 2019), we investigated if there was a similar pattern in morph-biased genes in *B. terrestris*. First, we examined if morph-biased genes had elevated rates of divergence by comparing the coding sequences (CDS) of homologous genes between *B. terrestris* and closely related bee species and for each comparison, calculating the ratio of nonsynonymous and synonymous substitutions (dN/dS). Higher dN/dS values indicate higher rates of structural divergence, which may reflect relaxed selection or in the case of excessive nonsynonymous mutations (dN/dS > 1), positive selection (Nielsen 2005). In contrast, low dN/dS values, which reflect excesses of synonymous mutations, suggest a gene is experiencing stronger selective constraints, consistent with purifying selection to conserve gene and associated protein function. Based on calculations of dN/dS with the closely related *Bombus impatiens* (supplementary table S4, Supplementary Material online), we found that genes with queen-biased expression in both nonreproductive tissues were more diverged between species compared to non-biased genes (Wilcoxon rank-sum test, Bonferroni-adjusted *P* < 1e-6; Fig. 2e and f), suggestive of relaxed constraints. In contrast, in the RTs, queen-biased genes showed significantly greater levels of conservation at the nucleotide level between bumblebee species compared to non-biased genes (Wilcoxon rank-sum test, Bonferroni-adjusted *P* = 8.1e-7; Fig. 2h). In the ovary, we did not find a significant difference between queen-biased and non-biased genes with both having low dN/dS values (Wilcoxon rank-sum test, Bonferroni-adjusted *P* = 1; Fig. 2g). Worker-biased genes showed significantly elevated dN/dS values compared to nonbiased genes in ovaries (Wilcoxon rank-sum test, Bonferroni-adjusted *P* = 0.0043; Fig. 2g). A similar pattern of queen-biased genes showing stronger selective constraints (lower dN/dS) in reproductive tissues was also found through comparisons with the other representative bee species found within the same genus (*Bombus polaris*) and family (*Apis mellifera* and *Habropoda laboriosa*; supplementary fig. S4, Supplementary Material online).

### Duplicated Genes Show Greater Tissue-specificity, More Relaxed Evolutionary Constraints and Differential Usage by Queens and Workers

**Fig. 3.**
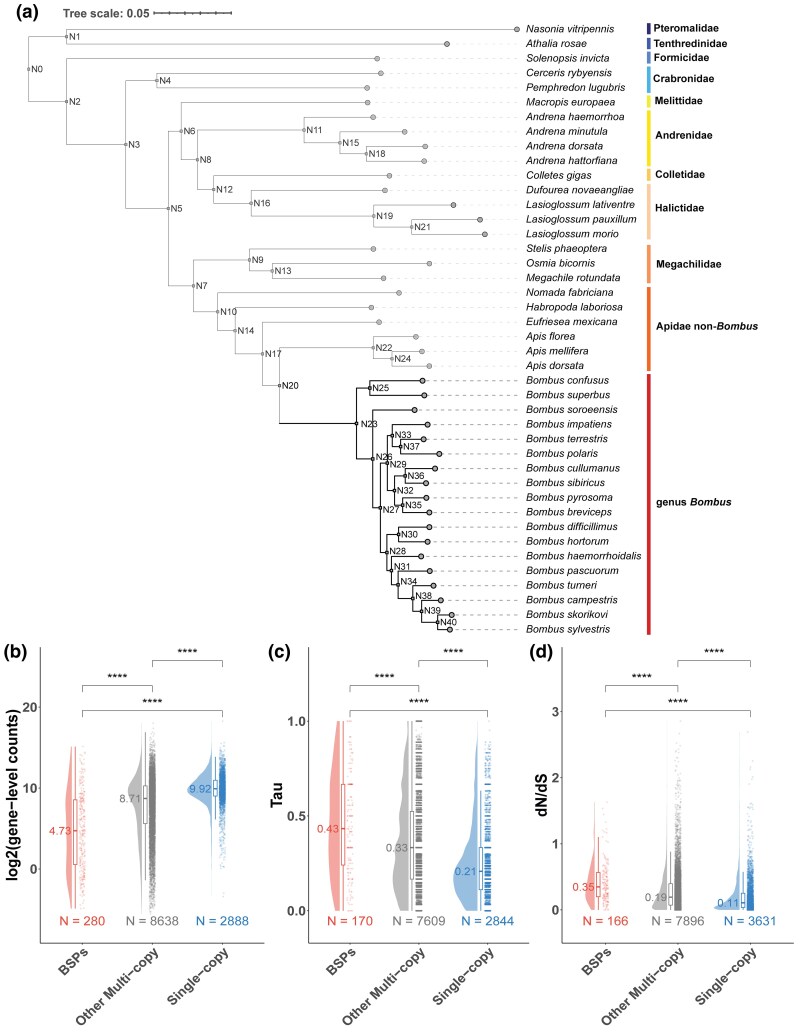
BSPs show lower gene expression, greater tissue-specificity, and greater evolutionary divergence rates. a) A phylogenetic tree informed by an OrthoFinder-based comparative analysis of predicted proteomes for 42 hymenopteran species, including representatives of 10 families, with the respective name of the family or genus for each species listed on the right hand side of the tree; b) raincloud plots displaying log2-transformed gene-level counts (*y* axis) for BSPs, other multicopy, and single-copy genes in bumblebees; c) raincloud plots displaying tissue-specificity (tau value) of BSPs, other multicopy, and single-copy identified in bumblebees; and d) Raincloud plots displaying dN/dS values calculated via pairwise comparisons of CDS between *B. terrestris* and *B. impatiens* for BSPs, other multicopy, and single-copy identified in bumblebees. For each raincloud plot, the “cloud” part represents the kernel density estimation of the data distribution, the “rain” part consists of individual data points (jittered for visibility). The median value for each respective measure per gene category is represented as a single line inside each box part with the actual median displayed on the left side of this line while the number of genes per gene category is shown below. For each pairwise comparison across gene categories, tests of significance with Bonferroni correction were performed using Wilcoxon rank-sum test with results shown above boxes representing the gene categories being compared (ns = not significant, * = Bonferroni-adjusted *P* < 0.05, ** = Bonferroni-adjusted *P* < 0.01, *** = Bonferroni-adjusted *P* < 0.001, and **** = Bonferroni-adjusted *P* < 0.0001).

Structural variation in the genome, such as gene duplication events, can give rise to functional novelty, yet their contribution to the expression of bumblebee morphs is largely unexplored. To understand how gene duplications may contribute to differences between bumblebee queens and workers, we first examined the presence of duplicated genes in the *B. terrestris* genome assembly. To do this, we performed a comparative genomic analysis involving 42 insect species, including 18 bumblebees, as well as representative members from 10 families across the Hymenoptera (Fig. 3a; supplementary table S5, Supplementary Material online). Using this approach, we identified a total of 348 sets of *Bombus*-specific paralogues (BSPs). While our analysis could have incorporated gene duplications that arose at an earlier evolutionary time-point or arisen solely within *B. terrestris*, the availability of genome assemblies for multiple bumblebee species, sampled across the genus, means that by using more than one assembly, we reduce the risk of identifying putative duplicated genes that are technical artifacts associated with assembly or annotation. Therefore, using duplicated genes conserved across multiple bumblebee species, we identified paralogous groups of genes (“sets”) that have two or more copies in the bumblebee species examined within our analysis compared to the hymenopteran species included as outgroups. In total, we identified 564 genes within our 348 BSP sets. To increase the confidence of subsequent analyses, we retained only BSPs located on the linkage groups, representative of chromosomes (based on the *B. terrestris* reference genome assembly, GCA_000214255.1; Sadd et al. 2015), leaving 175 sets of BSPs (288 genes; supplementary table S6, Supplementary Material online). These genes represent putative gene expansions within the bumblebee lineage with the majority (*n* = 192, 66.7% of all BSPs) predicted to have originated within the last common ancestor of *Bombus* species. While a considerable proportion of BSPs (*n* = 121, 42% of all BSPs) were annotated as uncharacterized, annotated BSPs consisted of 45 olfactory receptors and 12 cytochrome P450 family members, which form part of large gene families. In addition, genes coding for proteins related to energy metabolism, such as glucose dehydrogenase and peroxisomal acyl-coenzyme A oxidase, were also represented.

Before assessing whether BSPs differed in expression between queens and workers, we compared mean expression levels to other multicopy (i.e. non-BSP genes found in multiple copies (paralogues) across all species in our analysis; *n* = 8,638 genes) and single-copy genes (i.e. genes found in single copies across all species in our analysis, *n* = 2,888 genes). We found that BSPs, on average, had the lowest expression levels across all genes (median summarized gene-level count = 4.73; Wilcoxon rank-sum test, Bonferroni-adjusted *P* < 2.22e-16), including being less than half of the expression of single-copy genes (median summarized gene-level count = 9.92; Fig. 3b). This is a pattern consistent with previous research on younger gene duplicates in other species (Ma et al. 2024; Peng and Zhao 2024). As lower median expression in BSPs may be explained by greater variation in expression profiles across tissues, we next determined if BSPs were more tissue-specific. By comparing tau values for BSPs, multicopy and single-copy genes, we found that BSPs had, on average, higher tissue-specificity (Wilcoxon rank-sum test, Bonferroni-adjusted *P* < 1e-05), and were more than double that of single-copy genes, which were the most conserved in terms of expression across tissues (Fig. 3c). Lastly, we also found that BSPs exhibited significantly higher dN/dS values (Wilcoxon rank-sum test, Bonferroni-adjusted *P* < 1e-11) compared to other multicopy and single-copy genes, which suggests that BSPs may experience lower selective constraints (Fig. 3d).

**Fig. 4.**
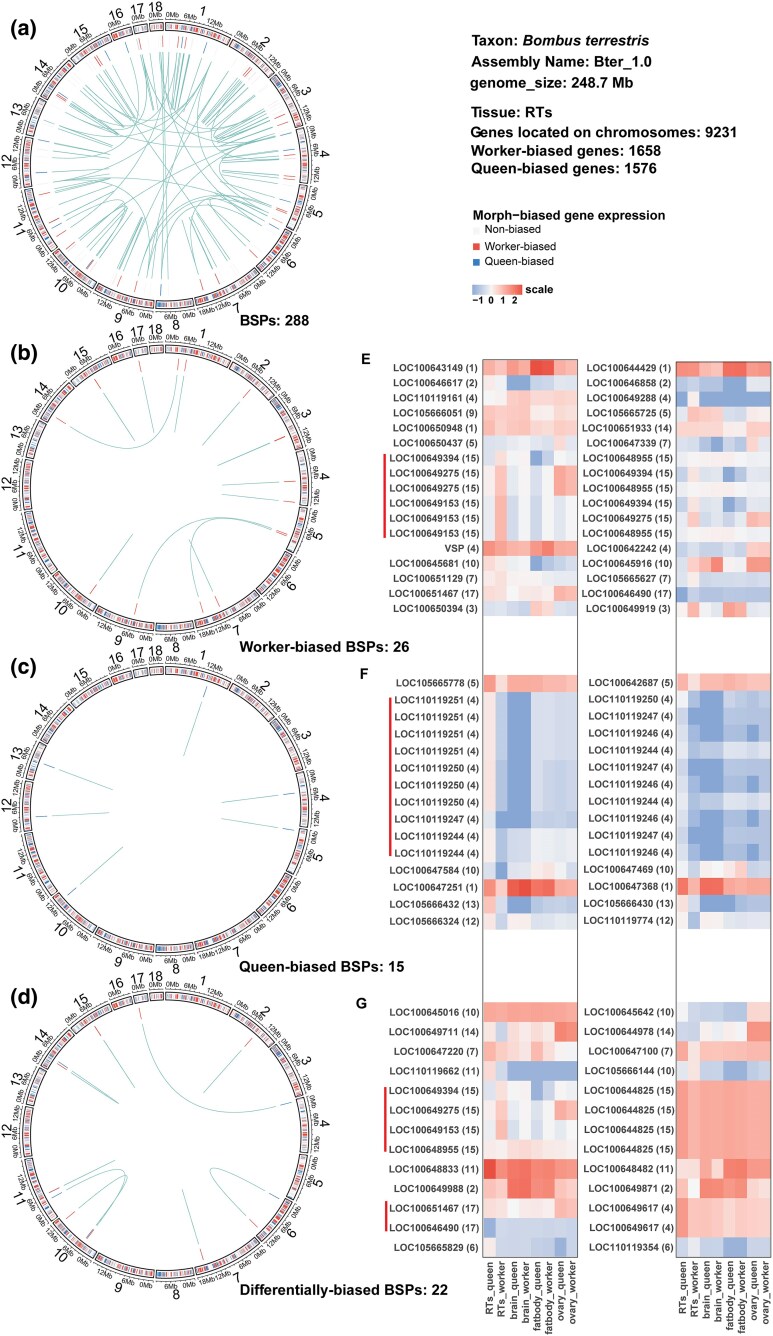
*Bombus*-specific gene duplicates are differentially expressed by queens and workers. a-d) Circos plots displaying: Layer 1: Individual blocks representing the relative size of the 18 chromosomal scaffolds of the *B. terrestris* reference genome assembly (Bter_1.0), with each colored, vertical band representing an individual gene, and the width of the band corresponding to the length of the gene; Layer 2: Each vertical band represents the physical location of an individual BSP with: a) all BSPs shown; b) only worker-biased BSPs shown; c) only queen-biased BSPs shown; and d) only differentially biased BSPs shown. Within the center of each Circos plot, the physical genomic location of BSPs within the same gene set are connected by lines, with the length of the lines determined by the distance between the BSPs; and e-g) heatmaps displaying gene expression in each tissue and morph for BSP pairs where all genes are: e) worker; f) queen; or g) differentially biased BSPs. The NCBI gene symbol is provided for each BSP, the physical chromosome of where the BSP is located is provided in brackets, while a line indicates pairs of genes that are in the same paralogous category (i.e. belong to the same BSP gene set).

To investigate whether certain BSPs differed in expression between social morphs, we examined morph-biased gene expression for BSPs in RTs, the tissue where we found the greatest difference in terms of the number of DEGs between bumblebee queens and workers. We found 12 sets of consistently worker-biased BSPs (i.e. duplicates that were significantly worker-biased; Fig. 4b), 6 sets of consistently queen-biased BSPs (i.e. duplicates that were significantly queen-biased; Fig. 4c), and 9 sets of differentially biased BSPs (i.e. duplicates where one copy showed significant worker-biased expression and the other copy showed queen-biased expression; Fig. 4d). Duplicated genes can experience divergent evolutionary trajectories (Lynch and Conery 2000), which may manifest in expression differences between duplicated pairs (Taylor and Raes 2004). To determine if paralogues within each set of BSPs differ in expression amplitude to each other, we calculated and compared gene expression levels for BSPs across tissues and morphs that showed consistent worker-biased, consistent queen-biased, and differentially biased expression, respectively. We found that the amplitude expression differences between queen-biased BSPs were significantly lower compared to BSPs exhibiting worker-biased (Wilcoxon rank-sum test, *P* = 0.00014) and differentially biased expression (Wilcoxon rank-sum test, *P* = 0.00023; Fig. 4e to g), indicating greater conservation in terms of expression profiles for BSPs with queen-biased expression.

In addition to divergence patterns in expression, duplicated copies may also experience differences in terms of structural rearrangements (Wyman et al. 2012). For the BSPs, we investigated the physical distribution of paralogues across the *B. terrestris* reference genome assembly. For this purpose, we first identified the physical location of each gene on individual chromosomal scaffolds (Fig. 4b to d; supplementary fig. S7, Supplementary Material online), finding that 151 BSPs (86% of all BSPs) were located on the same chromosome, a pattern found more often than random chance (Binomial exact test, *P* < 2.2e-16). We found that all queen-biased BSPs were located on the same chromosome as each other, with the distances between paralogues (median distance = 7,958 bp; minimum = 2,191 bp; maximum = 31,502 bp) being significantly lower (Wilcoxon rank-sum test, *P* = 0.0054) than the distances between differentially biased BSPs (median distance = 35,778 bp; minimum = 2,619 bp; maximum = 10,023,410 bp; supplementary table S6 and fig. S7, Supplementary Material online). Similar to the queen-biased genes, differentially biased BSPs were mostly, with the exception of two sets of BSPs, located on the same chromosome, which was also a similar pattern for worker-biased BSPs, although three had evidence of rearrangements post-duplication. Like queens, worker-biased BSPs were closer to each other than differentially biased genes (median distance = 12,789 bp; minimum = 2,470 bp; maximum = 95,610 bp; supplementary table S6 and fig. S7, Supplementary Material online). There was no significant difference (Wilcoxon rank-sum test: *P* = 0.4) in the distances of queen-biased BSPs compared to worker-biased BSPs (supplementary table S6 and fig. S7, Supplementary Material online).

## Discussion

The resolution of genomic conflicts at the molecular level can contribute to the expression of complex phenotypes that differ behaviorally, morphologically, and physiologically. Within social insects, the central role of differential gene expression within the expression of social morphs has been extensively examined while, in contrast, other mechanisms, such as gene duplication events, have received considerably less attention despite their ability to contribute to novel innovations. Using the large earth or buff-tailed bumblebee, a species with queens and workers that share a high level of anatomical similarity, we examined global and tissue-specific differences in gene expression between these social morphs across multiple tissues, as well as investigated the presence of duplicated genes that may contribute differently to the expression of queen and worker phenotypes. Through our transcriptomic-based analysis of four tissues, we find the largest differences in expression profiles across social morphs occur in the RTs, which includes the spermatheca, an organ, traditionally, deemed a vestigial structure in workers but recently, shown to be functional (Zhuang et al. 2023). In comparison, we find considerably less morph-biased genes in the brain, fat body, and ovary, which are tissues commonly studied for their contribution to social phenotypes, yet these genes exhibit higher levels of tissue-specificity. Furthermore, we find that morph-biased genes across tissues also differ in their level of evolutionary conservation with queen-biased genes showing patterns consistent with stronger selective constraints in reproductive tissues, including ovary and RTs. Lastly, we find evidence of differentially expressed gene duplications by queens and workers, indicating that duplicated genes may aid the expression of different social morphs.

As social insect morphs share the same genome, the expression of complex phenotypes is regulated by gene expression, which has led to differential gene expression in social insects being extensively investigated. A problem, however, is that previous studies, at least in bumblebees, have either been based on whole individuals (Colgan et al. 2011; Harrison et al. 2015), ignoring possible spatial or localized differences in gene expression, or have only explored differences in a single tissue (Ferreira et al. 2013; Lago et al. 2016; Lucas et al. 2017). To avoid interference of age and reproductive status with determining social morph differences, we analyzed queen and worker bees similar in age and stage of ovarian development, finding relatively moderate gene expression differences in tissues traditionally examined for and associated with queen and worker differences, such as the brain, fat body, and ovaries. Such morph-biased genes, however, had, on average, higher tissue-specificity compared to non-biased genes suggesting that: (i) queens and workers largely express a shared core transcriptome; and (ii) morph-biased genes are more localized and specialized in terms of where they are expressed, which may potentially allow or contribute to flexibility in the expression of the different morph phenotypes. Consequently, this may mean that such genes are important for expressing more subtle differences between morphs, which may underlie phenotypic differences associated with neurology, metabolism, and reproduction (supplementary table S7, Supplementary Material online). With the development of eusociality, the evolution of restricted and specialized genes may have increased to allow for more diverse functions arising from different morph divisions (Kapheim et al. 2015). For example, in the advanced eusocial honeybee *A. mellifera*, morph-associated genes show higher tissue-specificity (Warner et al. 2019), which is consistent with our findings, highlighting how restricted expression may potentially resolve conflict in the generation of novel phenotypes.

In comparison, the greatest differences in expression between social morphs were found in the spermatheca and associated tissues where, for example, compared to ovaries, we found 13 times more DEGs. Like many other social insect species, bumblebee workers can produce male-destined unfertilized, haploid eggs (Alaux et al. 2007), with high levels of ovarian activation occurring over the course of the colony life-cycle, especially during the “competition-point” whereby conflict over reproduction develops between the workers and the queen (Alaux et al. 2004). In addition, workers recently have been shown to be able to produce colonies after artificial insemination, to be able to mate, and also respond in a similar manner to insemination at the molecular level (Zhuang et al. 2023). Our analysis, here, highlights more intrinsic differences in the spermatheca and associated tissues between queens and workers, which may influence potential reproductive tactics. For example, like other social insect queens, bumblebee queens mate and can store sperm for a prolonged period, which is required as queens generally enter dormancy during winter periods in temperate climates, and establish colonies in the early spring. In contrast, our understanding of the biology and associated functionality of the worker spermatheca is limited but as evidence is lacking on the ability of workers to enter and survive dormant periods, workers may be unable to store sperm for extended periods, which may contribute to or explain differences identified at the transcriptional level. Regardless of the reason for these observed differences, the fact that such large differences were observed compared to other tissues highlights the spermatheca and associated tissues, namely the vagina and median oviduct, as particularly interesting organs in terms of understanding more fundamental differences between social morphs.

Within social insects, it is posited that selection will act relatively weaker on worker-biased traits in contrast to queen-biased traits where workers do not gain a fitness benefit from direct investment in their own reproduction, as well as in colonies where relatedness among nest-mates is low (Pennell et al. 2018). Indeed, morph-biased genes were previously found to have higher dN/dS values in social insects suggestive of directional selection acting on genes relevant to morph differentiation (Hunt et al. 2011, 2013; Lucas et al. 2017; Warner et al. 2019). Many bumblebee species are monandrous meaning that relatedness among workers is high, while, in addition, direct investment by workers in reproduction is evident, with ovarian activation and egg-laying relatively common observations, particularly, during the competition phase of the colony life-cycle (Alaux et al. 2007). Differences in reproduction have been traditionally one of the primary factors in distinguishing social insect morphs, with reproductive division of labor playing a central role in the evolution of phenotypic morphs. Indeed, within tissues associated with reproduction, we find evidence of slightly elevated dN/dS values in worker-biased genes, which may indicate that such genes are experiencing more relaxed selection compared to queen-biased genes.

Tissue-specificity has been proposed to evolve following gene duplications (Mantica et al. 2024) contributing to the generation of novel innovations, through the restricted expression of duplicated genes in specialized tissues (Hargreaves et al. 2014). Similarly, gene duplications have been proposed as playing an important role in the development of eusociality through the expansion of gene families (Chau and Goodisman 2017; Mikhailova et al. 2024). Our examination of morph-biased genes, which showed, on average, greater tissue-specificity, revealed that such genes consisted of more paralogous genes providing support for the contributory role of duplicated genes in the expression of social phenotypes. This finding was further supported by the identification and characterization of more recent *Bombus*-specific gene expansions, of which, some displayed signatures of differential usage between queens and workers.

To increase confidence in putative gene duplicates, we focused our analysis on paralogues found in the genome assemblies of extant bumblebee species (Sadd et al. 2015; Sun et al. 2021), including members of multiple subgenera. Given how assemblies were generated by separate research groups, employing different sequencing technologies and associated computational approaches, the identification of these duplications across multiple bumblebee genome assemblies provides greater confidence that such genes reflect true biological signals rather than assembly artifacts. Our homology-based analysis placed the origin of most duplication events at a period within the evolution of the Apidae, and, therefore, could be considered relatively young (median: 82 MYA from the split with a common ancestor with *A. mellifera*; Kumar et al. 2017). As these duplicated genes were also found in social parasitic bumblebees, which lack the worker morph, it may be suggested that such genes perform either a function additional to that commonly used by social morphs or possibly a novel function within parasitic bumblebees.

We found that BSPs had higher tissue-specificity and, on average, lower expression levels compared to single-copy orthologues and older paralogues. Collectively, this suggests that younger duplications in bumblebees are more restricted in terms of their expression levels and spatial distribution compared to older duplications. This may indicate that the importance and evolutionary potential of duplicated genes may increase over evolutionary time through the acquisition of additional or novel functions. Alternatively, the restricted site of expression may mean that such genes may be lost more quickly, a fate associated with many duplicated genes (Lynch and Conery 2000). Most BSPs did not differ in expression between queens and workers indicating a putative conserved function across bumblebee morphs. However, for BSPs that were differentially expressed, most were found in the spermatheca and associated tissues with evidence of BSPs with conserved expression biases in the queen or worker, respectively. Such BSPs generally showed lower differences in expression between pairs within a morph and were located closer to each other within chromosomes. In comparison, BSPs that differed in their direction of expression between morphs were generally located further away from each other within chromosomes. This trend is consistent with findings in *Drosophila* where it was found that the differences in expression between duplicated pairs are associated with their location within the genome (Wyman et al. 2012). Duplicated pairs from queen- and worker-biased BSPs are more conserved in terms of predicted protein function based on gene expression profiles, protein sequence similarity, and physical location, which may mean that retention of duplications contributes to the enhancement of certain physiological functions, while differentially biased BSPs may be more important with respect to morph-specific tasks. Such morph-specific tasks have been previously suggested to have evolved through the process of subfunctionalization (Corona et al. 2013), highlighting this mechanism as a means of generating novel phenotypes, as well as resolving potential intermorph genomic conflict (Pennell et al. 2018).

## Conclusion

The role of differential gene expression has long been acknowledged as a key component underlying complex phenotypic differences between social insect morphs yet direct comparisons have suffered from intrinsic differences, such as morphological differences, age, or reproductive status. Here, our transcriptomic-based analysis demonstrates the benefits of utilizing multiple tissues to understand global differences in gene expression patterns between bumblebee queens and workers, highlighting the importance of localized expression underlying phenotypic differences between bumblebee morphs. However, further insights into general morph differences, as well as the role of gene duplications, can be achieved through the profiling of more tissues. Similarly, while our study focuses on adults, additional studies of larvae at different instars would provide temporal support of expression differences, as well as copy number variant usage, between morphs, allowing us to explore morph determination and differentiation in more detail. Similarly, as sex-biased, particularly male-biased, genes generally show patterns of elevated rates of evolution (Ellegren and Parsch 2007), transcriptomic profiling of male tissues, which are generally understudied in the context of social insects (Baer 2014), will also be beneficial in helping to understand the importance of genes associated with female morph differences related to social behavior, given the males do not express the same behavioral repertoire of social interactions (although see Cameron 1985). Lastly, the reproductive tissues, especially the spermatheca, are interesting candidate tissues for further understanding gene expression between social morphs, given the potential role they contribute to in the evolution of alternative reproductive strategies displayed by certain social insects. Collectively, our study highlights how evolution can act at localized scales in terms of tissue-based expression and gene duplication events to contribute to the expression of social morphs, which, ultimately, contributes to advancing our understanding of the molecular mechanisms underlying the expression of complex phenotypes found in social insect colonies.

## Methods

### Sample Information

To examine transcriptional differences between female social morphs in *B. terrestris*, we analyzed publicly available transcriptomic data from queen and worker bumblebees. A previous study used this dataset to examine the gene expression profile changes in inseminated queens and workers finding similar patterns in social morph responses to insemination (Zhuang et al. 2023). Full details on sample collection and generation are provided by Zhuang et al. (2023) with an overview of sample sizes summarized in supplementary table S8, Supplementary Material online. In brief, the data consists of inseminated and noninseminated workers and queens sampled at three stages of ovarian development: stages I, II, and IV. For workers, bees were collected for each stage by sampling workers at days 3 (stage I), 4 (stage II), and 7 (stage IV) post-eclosion. For queens, bees were sampled at days 4 (stage I), 5 (stage II), and 7 (stage IV) post-eclosion. Therefore, the queens and workers consisted of all three ovarian developmental stages and both inseminated and noninseminated conditions, reducing the potential influence of differences in development and stage of reproduction, which can affect comparisons of gene expression between social morphs. The total dataset consists of 256 RNA-seq libraries collected from queens (*n* = 24) and workers (*n* = 48) for each of three tissues: brains, fat bodies, and ovaries. An additional fourth tissue sample consisted of other reproductive tissues (*n* = 20 queens, *n* = 20 workers), including the spermatheca, vagina, and median oviduct. For each library, sequencing (2 * 150 bp) was performed on an Illumina NovaSeq6000 platform with approximately 48.9 million reads generated per sample (Zhuang et al. 2023).

### Quality Assessment, Read Alignment, and Generation of Gene-Level Counts

We performed an initial quality assessment of sequencing data for each sample using FastQC (v.0.11.9) (Andrews 2019), which provided information on the potential presence and proportion of adaptor contaminants in reads, as well as low-quality bases. As quality was high, we did not perform filtering and moved directly to transcript quantification. For each sample, sequences were aligned using STAR (v.2.7.8a) (Dobin et al. 2013) against a *B. terrestris* reference genome assembly (Bter_1.0; GCA_000214255.1; Sadd et al. 2015) obtained from the Ensembl Metazoa database. During alignment, for each sample, we generated gene-level counts by using the parameter “–quantMode GeneCounts”. Lastly, for each STAR-generated alignment file, we used Qualimap2 (v.2.2.1) (Okonechnikov et al. 2016) to estimate mapping statistics with the resulting qualimap reports visualized using MultiQC (v.1.7) (Ewels et al. 2016).

To provide further confidence in our estimates of gene expression, we performed two complementary approaches to allow for comparison with counts generated by STAR. First, for each sample, we estimated transcript abundances using the quasi-aligner Salmon (v.1.4.0) (Patro et al. 2017) using two approaches: (i) the first approach used the Salmon-map method, which mapped the sequencing data directly to a predicted *B. terrestris* transcriptome; and (ii) a second approach, which used the Salmon-align method, involved the conversion of STAR-based alignments into transcript-coordinate BAM files using Mudskipper (v.0.1.0) (https://github.com/OceanGenomics/mudskipper). More specifically, we projected the genome-based alignment coordinates of each aligned sequence to transcript-based coordinates as described in a reference gene transfer file. Then, the transcript-coordinate BAM files were used to perform transcript abundance estimation using the Salmon-align method, which allowed for an examination of overlap between the expression estimates generated by STAR and Salmon. For both Salmon-map and Salmon-align methods, we used coding and noncoding transcript coordinates simultaneously extracted from the *B. terrestris* genome assembly using Bedtools (v.2.30.0) (Quinlan and Hall 2010). In addition to base parameters, we ran both Salmon methods using the “–gcBias” flag, which assists and trains the program to avoid fragment-level GC biases present in the input data. Salmon-based transcript abundance estimates generated by both methods were used for differential expression analysis.

### Differential Gene Expression Analysis to Determine Morph-Biased Genes

To assess differences in gene expression between bumblebee morphs, we first imported STAR-generated gene-level counts for all samples directly into RStudio. We next prefiltered gene-level counts to remove lowly expressed genes or genes with no detectable expression, which are not biologically informative. We removed genes with a sum of reads <10 across all samples. From all annotated genes present in the reference genome (*n* = 12,008), our filtering approach resulted in the retention of >93% of genes for downstream analyses (retained genes: reproductive tissues = 11,284; brain = 11,502; fat body = 11,370; ovary = 11,567). To first assess transcriptional profiles across tissues, we performed variance-stabilizing transformation of gene-level counts using DESeq2 (v.3.16) (Love et al. 2014). Using these transformed counts, we then performed a PCA, which revealed clustering of individual samples based on tissue of origin (Fig. 1a to c). Given the observed presence of tissue-biased profiles, we performed differential expression analysis between queens and workers for each tissue using likelihood-ratio tests (LRT) implemented in DESeq2. Each LRT consisted of a full model, which included “stage” (ovarian development stage), “condition” (inseminated/noninseminated), and “social morph” (queen/worker) and a reduced model, which included “stage” and “condition” only. With an LRT, we compared our full and reduced models to identify differences in expression, which were explained by our main effect, “social morph”, rather than the other terms (stage of ovarian development (“stage”) and insemination status (“condition”)), which were included as covariates in our analysis. We classified a gene as being differentially expressed between morphs if it had a Benjamini–Hochberg adjusted *P-*value (padj) of less than 0.05 and an absolute log2-fold change of equal to or greater than 1.

For transcript abundance estimates generated by both Salmon-map and Salmon-align, we first converted these values into gene-level counts using tximport (v.1.26.1) (Soneson et al. 2016). This conversion step was based on a transcript-to-gene map generated from the *B. terrestris* genome assembly. For each method, we then performed a differential gene expression analysis as previously outlined for the STAR-based gene-level counts. For comparison with STAR-based counts, we performed correlation-based analyses of the values of log2FoldChange calculated for all genes identifying relatively high correlations (Pearson's Correlation Coefficient *R* ≥ 0.9, *P* < 0.05) in expression across methods for each tissue (supplementary fig. S1, Supplementary Material online). We also compared DEGs identified through DESeq2 based on gene-level counts determined by STAR, Salmon-map, and Salmon-align methods, and found that at least 70% of DEGs were identified using all methods (supplementary fig. S1, Supplementary Material online).

### Tissue-Specificity Analysis

As social morphs may share a core transcriptome with, instead, morph-biased expression differences localized to certain tissues, we examined tissue-specificity with respect to expression for each gene across the four tissues. For this, we calculated for each gene a tissue-specificity value according to the tau (τ) algorithm (Yanai et al. 2005), which represents the best standard to recognize tissue-specific genes (Kryuchkova-Mostacci and Robinson-Rechavi 2017). We first normalized STAR-generated gene-level counts for each tissue using the transcripts per kilobase per million (TPM) method (Long et al. 2023). Genes with the lowest 10% mean expression across all samples, as measured in TPM values, were identified and removed from further analysis as such lowly expressed genes are biologically noninformative. To make genes across different tissues comparable, we first performed a log2-transformation to normalize our expression values using the tispec R package (v.0.99.0) (https://rdrr.io/github/roonysgalbi/tispec). Second, using tispec, for each gene, we calculated a tau value between 0 (nonspecific, global expression) and 1 (tissue-limited expression). To determine if worker- and queen-biased genes show different levels of tissue-specificity, we compared and visualized tau values across morph-biased and non-biased genes using individual pairwise Wilcoxon rank-sum tests with the function “stat_compare_means” from the R package “ggpubr” (https://doi.org/10.32614/CRAN.package.ggpubr). The statistical significance of each comparison was corrected for multiple testing using the Bonferroni correction method.

### Estimation of Divergence at Macro-Evolutionary Scales

As morph-biased genes may evolve under different selective pressures compared to the background genome, we investigated signatures of divergence at the macroevolutionary scale. For both morph-biased and nonbiased genes, we calculated the ratio of nonsynonymous (dN) to synonymous (dS) substitutions between the CDS of *B. terrestris* and homologous sequences in closely related bee species. We used the dN/dS values to assess the degree of selective constraints acting on morph-biased genes. For example, the lower the dN/dS value a gene has, the more evidence that a gene is evolving under stronger purifying selection while an excess of nonsynonymous to synonymous mutations is representative of positive selection. We calculated dN/dS through comparing the CDS of *B. terrestris* with those of the closely related *B. impatiens*. As an additional step, we also calculated dN/dS values for *B. terrestris* by comparing with another closely related bumblebee, *Bombus polaris*, the Western honeybee, *A. mellifera* (divergence time: 78MYA), and a solitary apid, *H. laboriosa* (divergence time: 108MYA). Entire CDS sequences of *B. impatiens*, *A. mellifera*, and *H. laboriosa* were obtained from Ensembl Metazoa (*B. impatiens*: GCA_000188095.4; *A. mellifera*: GCA_003254395.2; *H. laboriosa*: GCF_001263275.1) (Kapheim et al. 2015; Sadd et al. 2015; Wallberg et al. 2019), and whole CDS sequences of *B. polaris* was obtained from the Ensembl Rapid Release database (GCA_014737335.1; Sun et al. 2021). For *B. terrestris*, we extracted whole CDS sequences from its reference genome assembly using GffRead (v.0.12.7) (Pertea and Pertea 2020). The dN/dS values were calculated using the dNdS() function from the orthologr R package (v.0.4.2) (Drost et al. 2015). First, for each individual species comparison, DIAMOND2 (Buchfink et al. 2021) was used to align all CDS sequences. We next used the BLAST reciprocal best hit method to infer homologous sequences between the two species being compared (E-value cutoff of 1E-5). Using these alignments, dN/dS values were computed using the “Comeron” method (Comeron 1995). Using this approach, we inferred homologous sequence and calculated dN/dS values for 11,679, 10,029, 10,262, and 8,891 genes of *B. terrestris* based on comparisons with *B. impatiens*, *B. polaris*, *A. mellifera*, and *H. laboriosa*, respectively (supplementary table S4, Supplementary Material online). Similar to above, to investigate whether worker- and queen-biased genes display different levels of selective constraints, we compared median dN/dS values between morph- and nonbiased genes using individual pairwise Wilcoxon rank-sum tests with the function “stat_compare_means” from the R package “ggpubr” (https://doi.org/10.32614/CRAN.package.ggpubr). The statistical significance of each comparison was corrected for multiple testing using the Bonferroni correction method.

### Identification of Lineage-Specific Duplicated Genes

To determine duplicated genes in *B. terrestris*, we performed a homology-based analysis using OrthoFinder (v.2.5.4) (Emms and Kelly 2019) with the following species (*n* = 42): 18 bumblebee (*Bombus*) species, including representatives from each of the main subgenera, 6 additional species from the family Apidae, 13 additional species from other members of the clade Anthophila, as well as 5 additional species from the order Hymenoptera to serve as outgroups, including representatives of the wasps (*n* = 3), ants (*n* = 1), and sawflies (*n* = 1). The full list of species is provided in supplementary table S5, Supplementary Material online. For each species, we obtained the predicted proteomes from the Ensembl Metazoa and Ensembl Rapid Release databases. We ran OrthoFinder with the default parameters, which calculated protein sequence similarity values for all aligned protein pairs between species using DIAMOND. As part of this analysis, OrthoFinder grouped homologous sequences within and across species into evolutionary units, known as orthogroups. In addition, Orthofinder identified duplicated genes across lineages. Using these genes, we searched and subsetted putative BSPs, which include putative duplicated genes found across 18 bumblebee species (i.e. on the internal node of all extant bumblebees), up to the branch containing *B. terrestris*, which were used as the basis for examining patterns of differential expression between morphs.

### Assessment of Functional and Structural Divergence Between Duplicated Genes

To determine if BSPs show patterns in structural and putative functional divergence, we first investigated expression differences among paralogues. For each tissue, we examined if BSPs were differentially expressed between social morphs. Using this information, we next examined if duplicated genes had conserved morph biases in terms of expression (i.e. paralogues consistently showed queen- or worker-biased expression). Next, we examined if pairs differed in morph-biased expression (e.g. one pair was elevated in queens and the other pair in workers or vice versa), which may indicate evidence of subfunctionalization (Corona et al. 2013). In addition, as duplicated genes may evolve tissue-localized or restricted expression (Chau and Goodisman 2017), we examined if duplicated genes were more specific to certain tissues through examining tau values calculated for each gene. To further understand global expression profiles of BSPs across tissues and morphs, we subsequently performed a genome-wide-based clustering analysis using the R package DEGreport (v.1.34.0) (Pantano 2022). The clustering analysis was run using normalized gene-level counts generated by DESeq2. In addition, to investigate gene expression differences between paralogous pairs of BSPs across four tissues and two social morphs, we first calculated the mean normalized gene-level counts of each gene across all samples within each subset (i.e. a specific tissue-morph combination). Next, we quantified the expression difference between two genes in each paralogous pair within each subset by computing the absolute difference of their mean expression values. Finally, we summed these differences across all eight subsets (4 tissues × 2 social morphs). This value provided an overall estimate of expression differences between paralogous pairs across morphs and tissues with a larger value representing greater divergence in overall expression between pairs. Using all genes, we also performed a weighted gene coexpression network analysis using the R package WGCNA (v.1.72-5) (Langfelder and Horvath 2008; details of results are provided in Supplementary material).

To understand divergence at the structural level between paralogous pairs of BSPs, we first calculated the percentage of sequence similarity of the longest predicted protein isoform of each BSP using results generated by a DIAMOND-based analysis performed using OrthoFinder. Second, we extracted predicted protein domains for proteins encoded by each gene from Ensembl biomaRt (v.3.17) (Kinsella et al. 2011), and compared the number and types of domains per paralogous pair as variation in number and type may indicate differences in terms of protein structure and functionality. Lastly, we investigated the physical distance between duplicated genes, as genes in closer physical proximity may experience lower levels of functional and/or structural divergence compared to genes that are found over larger distances apart or on separate chromosomes (Wyman et al. 2012).

## Supplementary Material

msaf063_Supplementary_Data

## Data Availability

The scripts underpinning our analysis are publicly available at: https://github.com/hongfeifly/Bter_RNAseq_analysis. All the genome assemblies used for the comparative genomic analyses are publicly available on the Ensembl Metazoa and Ensembl Rapid Release databases, with the full list of genome assemblies provided in supplementary table S6. The RNA-seq data used in the present study are publicly available from the NCBI Sequence Read Archive database (BioProject Accession: PRJNA868857).
