# Supplementary material for: Localized Tissue-Specific Gene Expression and Gene Duplications are Important Sources of Social Morph Differences in a Social Bumblebee
Source: Mol Biol Evol. 2025 Mar 27;42(4):msaf063. doi: 10.1093/molbev/msaf063 (PMC11968646; doi:10.1093/molbev/msaf063)
Supplement: msaf063_Supplementary_Data [file msaf063_supplementary_data.zip › Supplementary material_MBE-24-0997.R1.pdf]

Supporting Information for:

Localised tissue-specific gene expression and gene duplications are important sources of social morph differences in a social bumblebee.

Hongfei Xu[1]* & Thomas J. Colgan[1,2]*.

1. Institute of Organismic and Molecular Evolution, Johannes Gutenberg University Mainz, Hanns-Dieter-Hüsch-Weg 15, 55128 Mainz, Germany.
2. Institute of Quantitative and Computational Biosciences (IQCB), Johannes Gutenberg University Mainz, Hanns-Dieter-Hüsch-Weg 15, 55128 Mainz, Germany.

Corresponding authors:

*Hongfei Xu

Email: honxu@uni-mainz.de

*Thomas J. Colgan

Email: tcolgan@uni-mainz.de

This PDF file includes:

Supporting text

Supplementary figures S1 to S10

SI References

Other supporting materials for this manuscript include the following:

Supplementary tables S1 to S8

Supporting Information Text

Gene-level count between social morphs

To further examine the difference in gene expression levels between female morphs in each tissue, we calculated the mean gene-level count for each gene per tissue in bumblebee workers and queens, respectively. Due to the huge span of values for gene-level counts, we first performed a log2 transformation on these counts, and then performed a correlation test, finding that log2-transformed mean gene-level counts were strongly positively correlated (Pearson's correlation coefficient, $R >= 0.9$) between workers and queens within each tissue (supplementary fig. S1 *A-D*). We then compared the mean gene-level counts across all samples for worker-biased, queen-biased and non-biased genes, finding that morph-biased genes were significantly lower for three of the tissues (Wilcoxon rank-sum test, $P < 0.05$; worker-biased: brain = 5.16, fat body = 6.76, ovary = 4.51; queen-biased: brain = 8.2, fat body = 7.49, ovary = 8.34) than non-biased genes (brain = 9.16, fat body = 9.23, ovary = 9.17; supplementary fig. S1 *E-H*). In comparison, in RTs, queen-biased genes showed significantly higher (Wilcoxon rank-sum test, $P <$ 1e-13) expression levels compared to both worker- and non-biased genes, with no significant difference (Wilcoxon rank-sum test, $P = 0.22$) between the latter two sets of genes (supplementary fig. S1). In addition, we found that the expression levels of queen-biased genes were significantly higher (Wilcoxon rank-sum test, $P <$ 1e-7) than those of the worker-biased genes in three tissues with the exception of the fat body (supplementary fig. S1).

Comparison of differential expression analyses based on STAR-output and salmon-output

To increase confidence in our results, we compared the results of our differential expression analysis obtained by STAR, with two transcriptome-based methods: Salmon-map and Salmon-align. First, we found a high positive correlation between gene-level counts generated by each of the three approaches (Pearson's Correlation Coefficient, $R = 0.96 \pm 0.04$; supplementary fig. S2).

Comparison of the morph-biased differentially expressed genes (Likelihood Ratio Test: Benjamini-Hochberg-adjusted $P < 0.05$) identified by each of the three methods also yielded a high percentage of overlap across each tissue: brain (80% overlap); fat body (78% overlap); ovary (80% overlap); and RTs (70% overlap; supplementary fig. S2).

Comparison of differential expression analyses between non-inseminated and all samples

Our analysis included inseminated and non-inseminated queens and workers as generated by Zhuang et al. (2023), which we incorporated into our analysis by including insemination status (condition) into our models as part of the differential expression analysis. As a complementary step, we compared differential expression patterns within inseminated and non-inseminated samples of queens and workers independently to further determine whether morph differences may be influenced by the inclusion of individuals that differed in insemination status. Using only non-inseminated queens and workers, we performed a differential expression analysis using STAR-based counts. We found a very high positive correlation (Pearson's correlation coefficient, $R = 0.98$) in terms of log2 fold change between non-inseminated samples and all samples, which forms the basis of our main analysis (supplementary fig. S3). In addition, using only the non-inseminated samples, we identified a total of 3,716 morph-biased differentially expressed genes (LRT: BH-adjusted $P < 0.05$), which shared a high overlap with morph-biased genes identified by our larger analysis (n = 3,470 shared DEGs; percentage of overlap = 88.01%; supplementary fig. S3).

dN/dS estimated in other species divergence node

To investigate if morph-biased genes evolve under more relaxed selection compared to non-biased genes in *B. terrestris*, we calculated dN/dS for such genes by comparing the coding sequences (CDS) of each gene between *B. terrestris* with four closely related bee species representing comparisons at different evolutionary scales of divergence. In addition to the eastern

common bumblebee *B. impatiens*, we selected another bumblebee *B. polaris* that is the closest relative to *B. terrestris* based on our orthofinder-inferred phylogenetic tree. At this species divergence node, based on a comparison of dN/dS between morph- and non-biased genes, we found a similar scenario to *B. impatiens*, whereby queen-biased genes showed significantly lower (Wilcoxon rank-sum test, $P < 0.001$) selective constraints compare to non-biased genes in non-reproductive tissues, such as the brain and fat body (supplementary fig. S4 *A* and *B*). In contrast, in RTs, queen-biased genes showed significantly higher (Wilcoxon rank-sum test, $P = 1.9e\text{-}6$) selective constraints as evidenced by lower dN/dS values compared to non-biased genes (supplementary fig. S4 *D*). In addition, worker-biased genes showed significantly lower (Wilcoxon rank-sum test, $P < 0.05$) selective constraints in the brain and ovary compared to non-biased genes (supplementary fig. S4 *A* and *C*). The two remaining species were selected from outside the genus *Bombus*, the Western honeybee *A. mellifera* and a solitary apid *H. laboriosa*. At these two nodes, we found that queen-biased genes showed significantly lower dN/dS (Wilcoxon rank-sum test, $P < 0.05$) compared to non-biased genes in both reproductive tissues (supplementary fig. S4).

Examination of selection acting on morph-biased genes at the population level

As positive selection can also be detected at the population level, we investigated genomic signatures of recent selection for each gene using measures of |nSL| generated by a previous population genomics study on wild-caught *B. terrestris* (Colgan et al. 2022). Higher |nSL| scores represent a greater likelihood of extended haplotype homozygosity and, therefore, of recent or ongoing selection, with |nSL| scores greater than two suggested as being evidence of recent positive selection (Ferrer-Admetlla et al. 2014; Szpiech and Hernandez 2014). Across tissues, we found variations in signatures of recent selection: for the fat body and RTs, |nSL| scores were significantly higher (Wilcoxon rank-sum test, $p < 1e\text{-}06$) for both worker-biased (median |nSL| score: fat body = 1.97; RTs = 1.79) and queen-biased (median |nSL| score: fat body = 2.09; RTs

= 1.83) genes compared to non-biased genes (median |nSL| score: fat body = 1.65; RTs = 1.61; supplementary fig. S5 *B* and *D*). For both tissues, morph-biased genes contained a significantly higher (Chi-squared tests: $P < 1e\text{-}06$) percentage of genes with |nSL| score greater than 2. In the ovary, queen-biased genes (median |nSL| score = 2.04) had significantly higher (Wilcoxon rank-sum test, $P < 0.01$) |nSL| scores than worker-biased (median |nSL| score = 1.74) and non-biased (median |nSL| score = 1.67) genes (supplementary fig. S5 *C*), while the percentage of genes with |nSL| scores greater than 2 was also significantly higher (Chi-squared test, $X^2 = 12.80$, DF = 2, $P = 0.0017$). In the brain, the |nSL| scores of queen-biased genes (median |nSL| score = 1.90) were significantly higher (Wilcoxon rank-sum test, $P = 0.04$) than non-biased (median |nSL| score = 1.67) genes (supplementary fig. S5 *A*), although there was no significant difference (Chi-squared test, $X^2 = 2.007$, DF = 2, $P = 0.37$) in the percentage of genes with |nSL| score greater than 2.

We also examined *Bombus*-specific paralogues (BSPs) for signatures of recent selection, finding that while BSPs did not differ significantly (Wilcoxon rank-sum test, $P > 0.05$; median |nSL| scores for BSPs = 1.62) from other multi-copy (paralogues) (median |nSL| = 1.71) or single-copy (median |nSL| = 1.59) genes in terms of |nSL| scores (supplementary fig. S6), they showed a significantly lower percentage of genes with |nSL| score > 2 than other multi-copy genes (Chi-squared test, $X^2 = 4.72$, DF = 1, $P = 0.03$) indicating that BSPs, on average, may be evolving under different forms of selection at the population level.

GO terms enrichment

We performed Gene Ontology (GO) term enrichment analyses for morph-biased and duplicated genes using the R package topGO (v.2.50.0) (Alexa and Rahnenfuhrer 2009). Due to the shortage of functional information for genes in the *B. terrestris* reference genome assembly, we obtained GO terms for homologous genes in the model organism *Drosophila melanogaster* via Ensembl Metazoa BioMarts (Kinsella et al. 2011) and then assigned them to their *B. terrestris* homologues, which were also obtained through BioMarts. We ranked all genes by log2FC informed by our

morph-biased differential expression analysis and performed GO term enrichment analyses for the ranked genes using the ‘weight01’ algorithm and rank-based Kolmogorov-Smirnov (KS) tests (node size = 20; $P < 0.05$). In comparison, for assessing functional enrichment of BSPs, we identified the enrichment of GO terms based on the ‘weight01’ algorithm and Fisher's exact test (node size = 20; $P < 0.05$).

To identify the biological processes (BP), cellular components (CC) and molecular functions (MF) that our target genes may be involved in, we performed GO term enrichment analyses for morph-biased genes in each tissue (supplementary table S3). In the RTs, we found significant enrichment (KS test: $P < 0.05$) of 35 BP, 12 CC and 16 MF GO terms in worker-biased genes, with the most strongly enriched terms in each of the three respective GO term categories being ‘mitochondrial translation’ (GO_0032543), ‘RNA polymerase II, holoenzyme’ (GO_0016591), and ‘nucleic acid binding’ (GO_0003676), respectively. We found significant enrichment (KS test: $P < 0.05$) for 55 BP, 11 CC and 16 MF GO terms in queen-biased genes with ‘ribonucleoside triphosphate biosynthetic process’ (GO_0009201), ‘cell cortex’ (GO_0005938) and ‘GTP binding’ (GO_0005525) being the most significant terms enriched in each respective category.

In the brain, we found 19 BP, four CC and 12 MF GO terms were significantly enriched (KS test: $P < 0.05$) in worker-biased genes. The most strongly enriched terms in BP, CC, and MF were ‘mitochondrial transmembrane transport’ (GO_1990542), ‘obsolete integral component of mitochondrial membrane’ (GO:0032592), and ‘proton transmembrane transporter activity’ (GO:0015078), respectively. For queen-biased genes, we found significant enrichment (KS test: $P < 0.05$) of 83 BP, nine CC and 17 MF GO terms with ‘cell adhesion’ (GO:0007155), ‘integral component of membrane’ (GO_0016021), and ‘structural constituent of chitin-based cuticle’ (GO_0005214) being the most significant terms enriched in each respective category.

In the fat body, we found 36 BP, 22 CC and 21 MF GO terms were significantly enriched (KS test: $P < 0.05$) in worker-biased genes, with the most strongly enriched terms in each of the three

categories being 'cell-cell adhesion' (GO_0098609), 'integral component of membrane' (GO_0016021), and 'calcium channel activity' (GO_0005262), respectively. For queen-biased genes, we found significant enrichment (KS test: $P < 0.05$) of 41 BP, nine CC and 16 MF GO terms, and the most strongly enriched terms in each category were 'fatty acid metabolic process' (GO_0006631), 'mitochondrial respirasome' (GO_0005746), and 'hydrolase activity, acting on ester bonds' (GO_0016788), respectively.

In the ovary, we found significant enrichment (KS test: $P < 0.05$) of 29 BP, five CC and 15 MF GO terms in worker-biased genes, with the most strongly enriched terms in each of the three categories being 'axoneme assembly' (GO_0035082), 'integral component of plasma membrane' (GO_0005887), and 'hexosyltransferase activity' (GO_0016758), respectively. For queen-biased genes, we found significant enrichment (KS test: $P < 0.05$) of 43 BP, nine CC and nine MF GO terms with 'protein-DNA complex assembly' (GO_0065004), 'integral component of plasma membrane' (GO_0005887), and 'gated channel activity' (GO_0022836) being the most significant terms enriched in each respective category.

In addition, we also performed functional enrichment analysis for BSPs (supplementary table S3). Through this analysis, we found that BSPs were significantly enriched (Fisher's exact test: $P < 0.05$) for 38 BP-, 25 CC-, and 16 MF-associated terms. The most strongly enriched terms in each of the three categories were 'sensory perception of smell' (GO_0007608), 'extracellular space' (GO_0005615) and 'flavin adenine dinucleotide binding' (GO_0050660), respectively.

WGCNA analysis for BSPs

To further examine gene expression profiles of BSPs that differ between bumblebee workers and queens, we performed a weighted gene co-expression network analysis (WGCNA) for the spermatheca and associated tissues using the R package 'WGCNA' (v.1.72-5; Langfelder, 2008). We used gene-level counts as input, which were transformed through variance stabilizing

transformation (VST, using a function provided by the R package DESeq2). Using these normalised counts, for each tissue, we, first, performed sample clustering using the base R function 'hclust' and removed possible outlier samples, where applicable. We then generated an adjacency matrix using the soft threshold power (β) determined by the functions pickSoftThreshold and scaleFreePlot from the 'WGCNA' package. The adjacency matrix was then transformed into a topological overlap matrix (TOM) followed by a topological dissimilarity matrix (1-TOM). Subsequently, we clustered genes into different eigengenes modules (minClusterSize = 30; deepSplit = 2) using the 'hclust' function and then combined highly correlated modules (cutHeight = 0.25). Finally, we generated correlation matrices between module eigengenes and variables of interest, such as female morph, helping us to examine whether BSPs were assigned to the modules, which were correlated with morph differences.

All genes that expressed in RTs clustered into six modules, including one module ("turquoise", n = 8850 genes) that was strongly positively correlated with social morph ("turquoise", R = 0.98, $P < 2.2e\text{-}16$) and three modules ("brown", n = 600 genes; "blue", n = 671 genes; "black", n = 91 genes) that were strongly negatively correlated with social morph ("brown", R = 0.75, $P < 2.2e\text{-}16$; "blue", R = 0.71, $P < 2.2e\text{-}16$; "brown", R = 0.83, $P < 2.2e\text{-}16$; supplementary fig. S7). Within respect to the BSPs, we found the six modules contained a total of 200 BSPs, from which, 151 BSPs were assigned to the "turquoise" module, and 39 BSPs were assigned collectively to the three negatively correlated modules ("brown", n = 10 BSPs; "blue", n = 18 BSPs; "black", n = 11 BSPs; supplementary fig. S8).

Duplicated genes largely retain structural integrity

Duplicated genes may experience divergent evolutionary trajectories that may manifest in structural differences between BSPs, including in the amino acid composition of their products. We found a strong correlation of pairwise protein sequence similarity (Pearson's Correlation Coefficient, $R = 0.97$, $P < 2.2e\text{-}16$) between BSPs (supplementary fig. S9). The percentage of

sequence similarity value ranged from 35 to 100%, which could represent the structural differences between pairwise BSPs based on low levels of reciprocal sequence similarity but also there were pairs that diverged across the 95% percentile, which may be associated with functional divergence.

We next compared the types and number of predicted protein domains contained with BSPs, as if duplicated genes experience structural and associated functional divergence, it may be predicted that paralogues would differ both in the type and number of protein domains they contain. Among the 173 sets of BSPs identified, the majority (n = 114, 65.8% of all BSPs) were identical in terms of both the types and number of protein domains (supplementary table S7). In contrast, only three sets differed in type (i.e., no overlap in predicted domains) while approximately a quarter (n = 40) differed in the number of protein domains. Sixteen sets had no specific protein domains found (supplementary table S7).

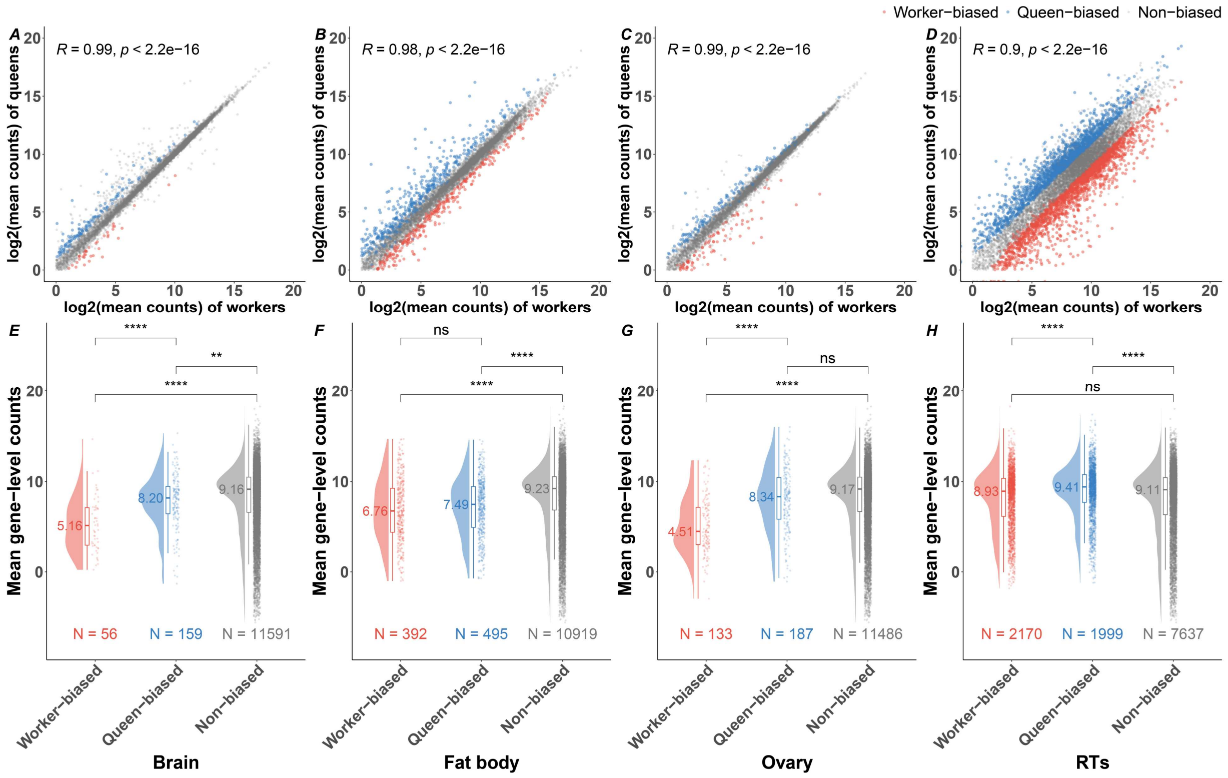

Supplementary figure S1. Spatial variation in expression profiles across social morphs in bumblebees. A-D) Scatter plots displaying the correlation (Pearson's correlation coefficient) of gene-level counts between queens and workers. Red dots represent worker-biased genes, blue dots represent queen-biased genes, and grey dots represent non-biased genes. The log2-transformed mean gene-level counts in workers and queens are provided on the x-axis and y-axis, respectively; and E-H) Raincloud plots showing the comparison of gene-level counts of worker-biased (red), queen-biased (blue) and non-biased (grey) genes in each tissue (brain, fat body, ovary, and RTs). Log2-transformed mean gene-level counts are provided on the y-axis. The "cloud" part represents the kernel density estimation of the data distribution, the "rain" part consists of individual data points (jittered for visibility). The median counts for each set are shown on the left side of the box. The number of genes in each set is shown below each corresponding plot. Tests of significance for differences in gene expression level between gene categories were performed using Wilcoxon rank-sum tests, the results, of which for pairwise comparisons with

Bonferroni correction are shown (ns = not significant, * = Bonferroni adjusted $P < 0.05$, ** = Bonferroni adjusted $P < 0.01$, *** = Bonferroni adjusted $P < 0.001$, **** = Bonferroni adjusted $P < 0.0001$).

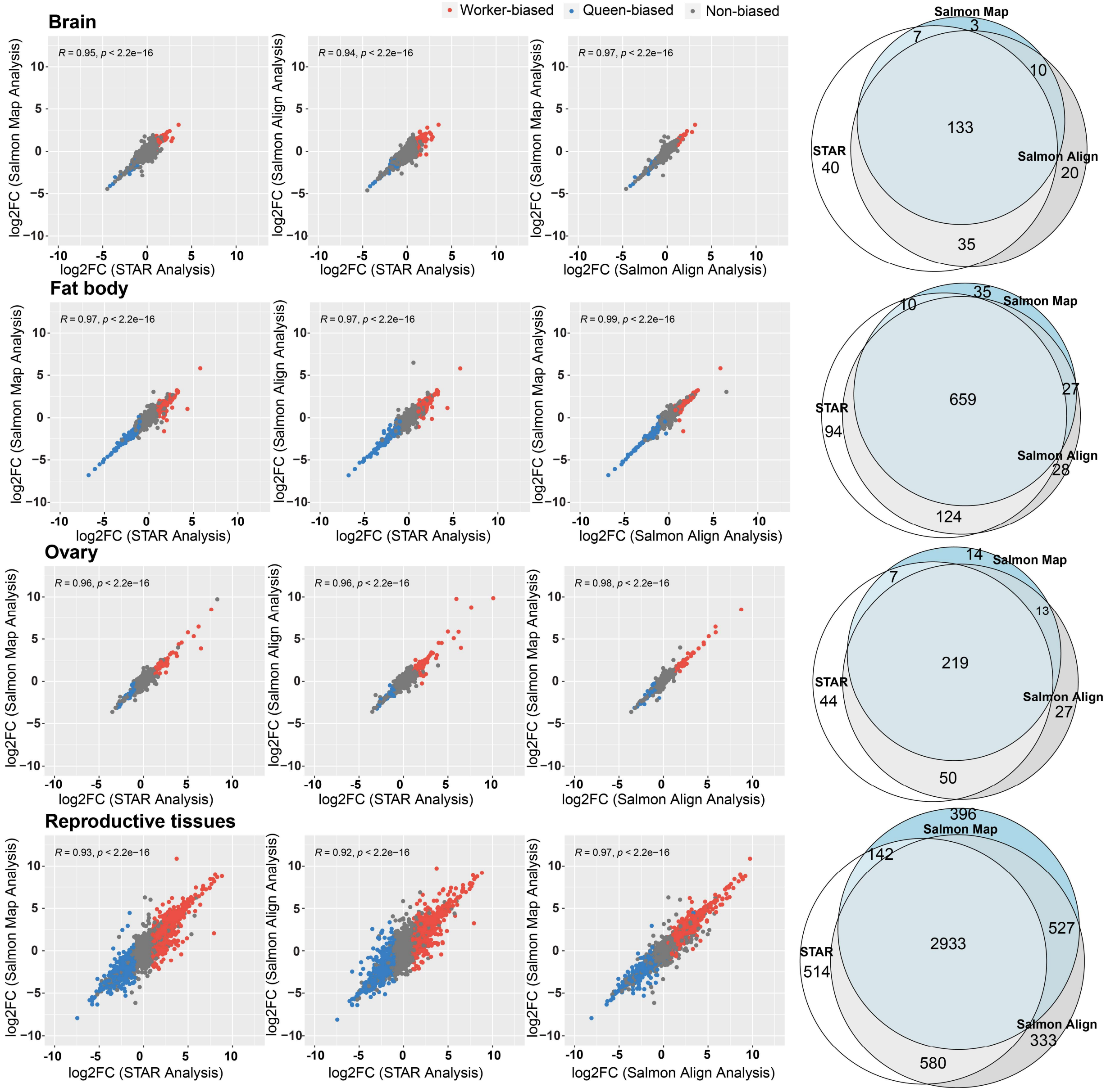

Supplementary figure S2. Comparisons of differentially expressed genes as identified on different methods. Scatter plots showing correlation (Pearson's correlation coefficient) of gene expression (log2FoldChange) from STAR-output, salmon-map output and salmon-align output in each tissue; and for each tissue, Euler plots displaying overlap in DEGs of STAR-output, salmon-map output and salmon-align output.

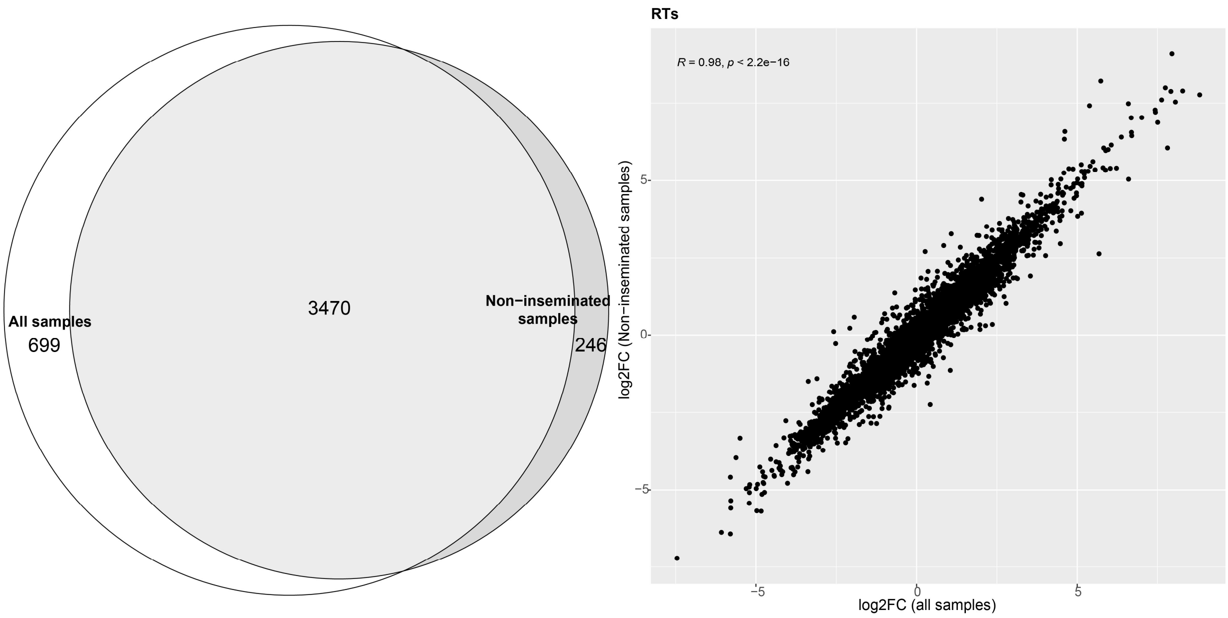

Supplementary figure S3: Comparison of differentially expressed genes between control and all samples. Euler plot displaying overlap in DEGs of all samples and non-inseminated samples in reproductive tissues; and Scatter plot showing correlation (Pearson's correlation coefficient) of gene expression (log2FoldChange) calculated by all samples and non-inseminated samples in reproductive tissues.

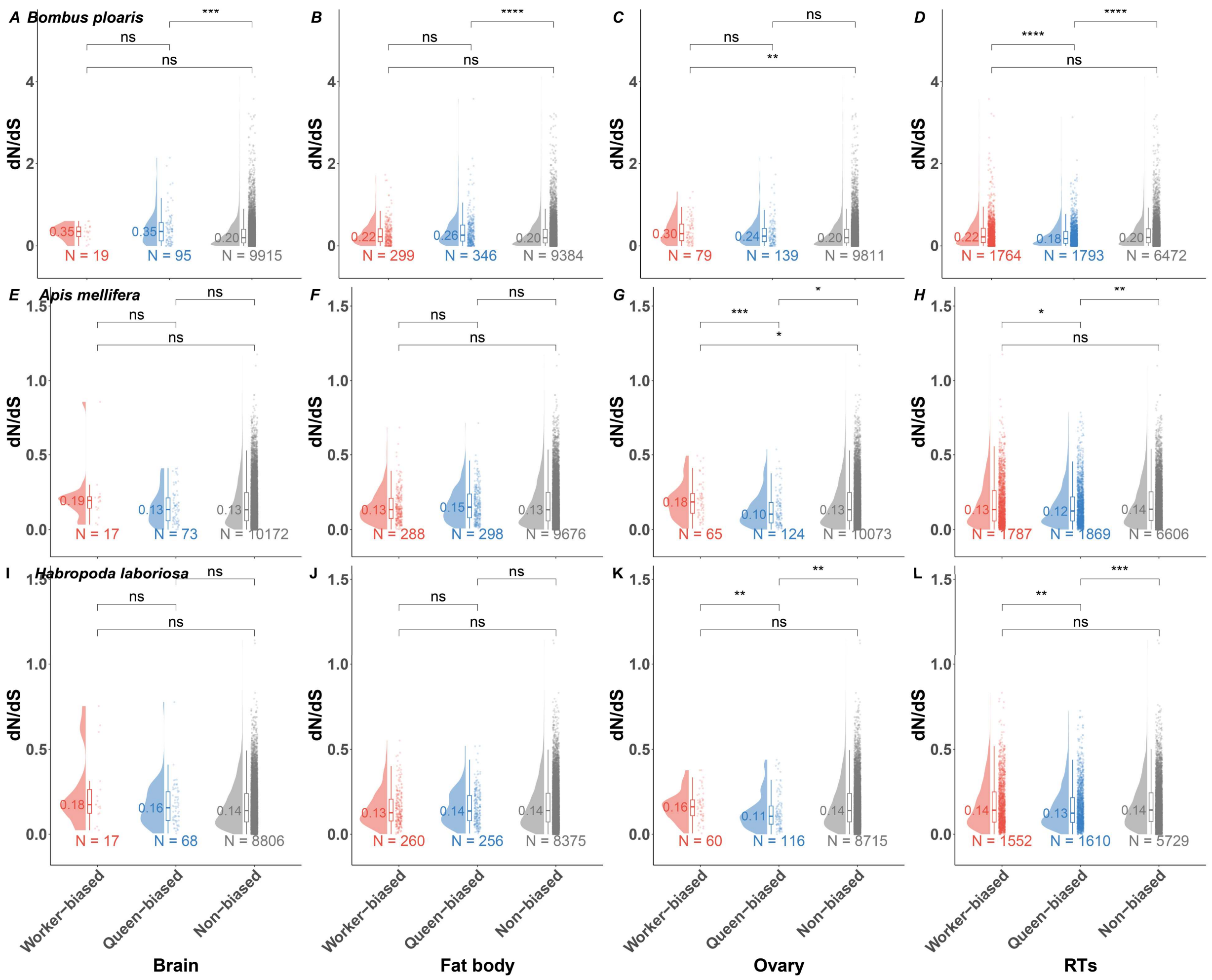

Supplementary figure S4: Comparisons between morph- and non-biased genes at different evolutionary scales of divergence. Raincloud plots displaying estimates of dN/dS values generated from comparing coding sequences between *B. terrestris* and the closely related *B. polaris* (same genus), *A. mellifera* (same family), and *H. laboriosa* (same family), for the worker- (red), queen- (blue) and non-biased (grey) genes for each tissue (brain, fat body, ovary, and RTs). For each raincloud plot, the "cloud" part represents the kernel density estimation of the data distribution, the "rain" part consists of individual data points (jittered for visibility). The median value for each set is shown on the left side of each box with the number of genes per set shown below the corresponding plot. For each pairwise comparison across gene categories, tests of significance with Bonferroni correction were performed using Wilcoxon rank-sum test with results

shown above plots representing the gene categories being compared (ns = not significant, * = Bonferroni adjusted $P < 0.05$, ** = Bonferroni adjusted $P < 0.01$, *** = Bonferroni adjusted $P < 0.001$, **** = Bonferroni adjusted $P < 0.0001$).

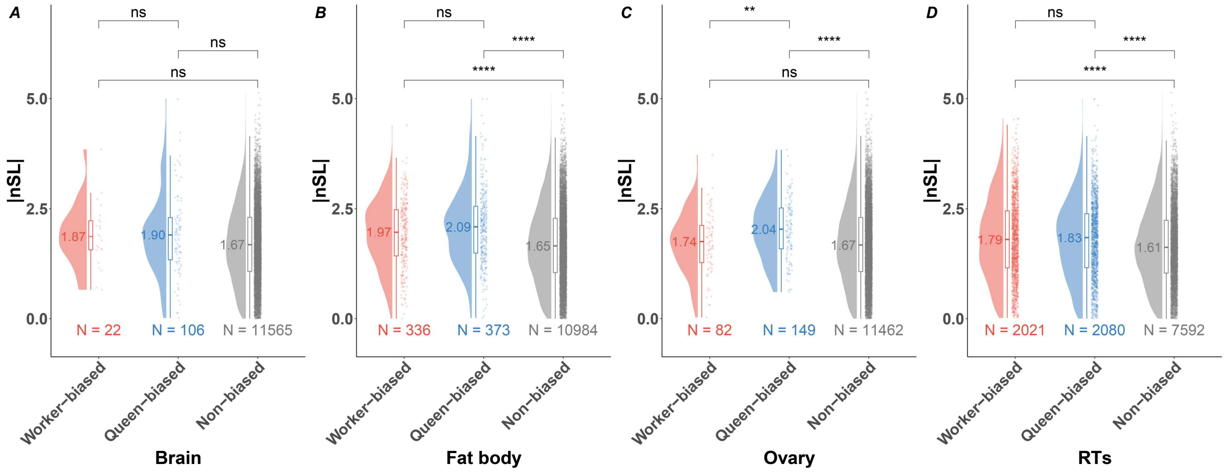

Supplementary figure S5: Recent or ongoing signatures of selection in morph-biased genes. A-D) Comparison of |nSL| scores ("number of segregating sites by length", which were used to predict recent selection) for the worker- (red), queen- (blue) and non-biased (grey) genes in each tissue (brain, fat body, ovary, and RTs). For each raincloud plot, the "cloud" part represents the kernel density estimation of the data distribution, the "rain" part consists of individual data points (jittered for visibility). The median |nSL| score per gene category is shown on the left side of each box. The number of genes per category is shown below the corresponding plot. For each pairwise comparison across gene categories, tests of significance with Bonferroni correction were performed using Wilcoxon rank-sum test with results shown above plots representing the gene categories being compared (ns = not significant, * = Bonferroni adjusted $P < 0.05$, ** = Bonferroni adjusted $P < 0.01$, *** = Bonferroni adjusted $P < 0.001$, **** = Bonferroni adjusted $P < 0.0001$).

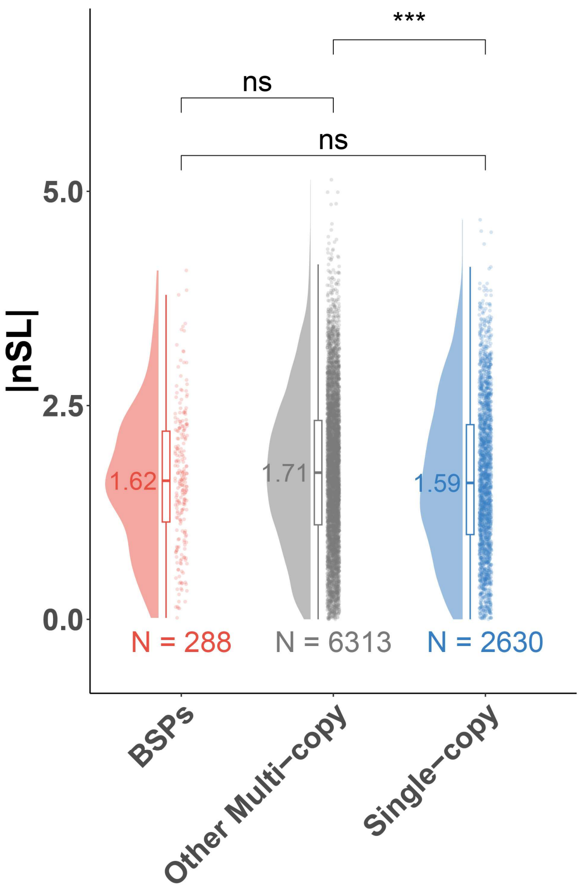

Supplementary figure S6: Recent or ongoing signatures of selection in *Bombus*-specific paralogues. Comparison of |nSL| scores ("number of segregating sites by length", which indicate patterns of recent or ongoing selection with increasing |nSL| scores) for BSPs (red), single-copy (blue) and other multi-copy (grey) genes identified in bumblebees. The "cloud" part represents the kernel density estimation of the data distribution, the "rain" part consists of individual data points (jittered for visibility). The median score for each gene category is shown on the left side of each box and the number of genes per gene category is shown below the corresponding plot. For each pairwise comparison across gene categories, tests of significance with Bonferroni correction were performed using Wilcoxon rank-sum test with results shown above plots representing the gene categories being compared (ns = not significant, * = Bonferroni adjusted $P < 0.05$, ** = Bonferroni adjusted $P < 0.01$, *** = Bonferroni adjusted $P < 0.001$, **** = Bonferroni adjusted $P < 0.0001$).

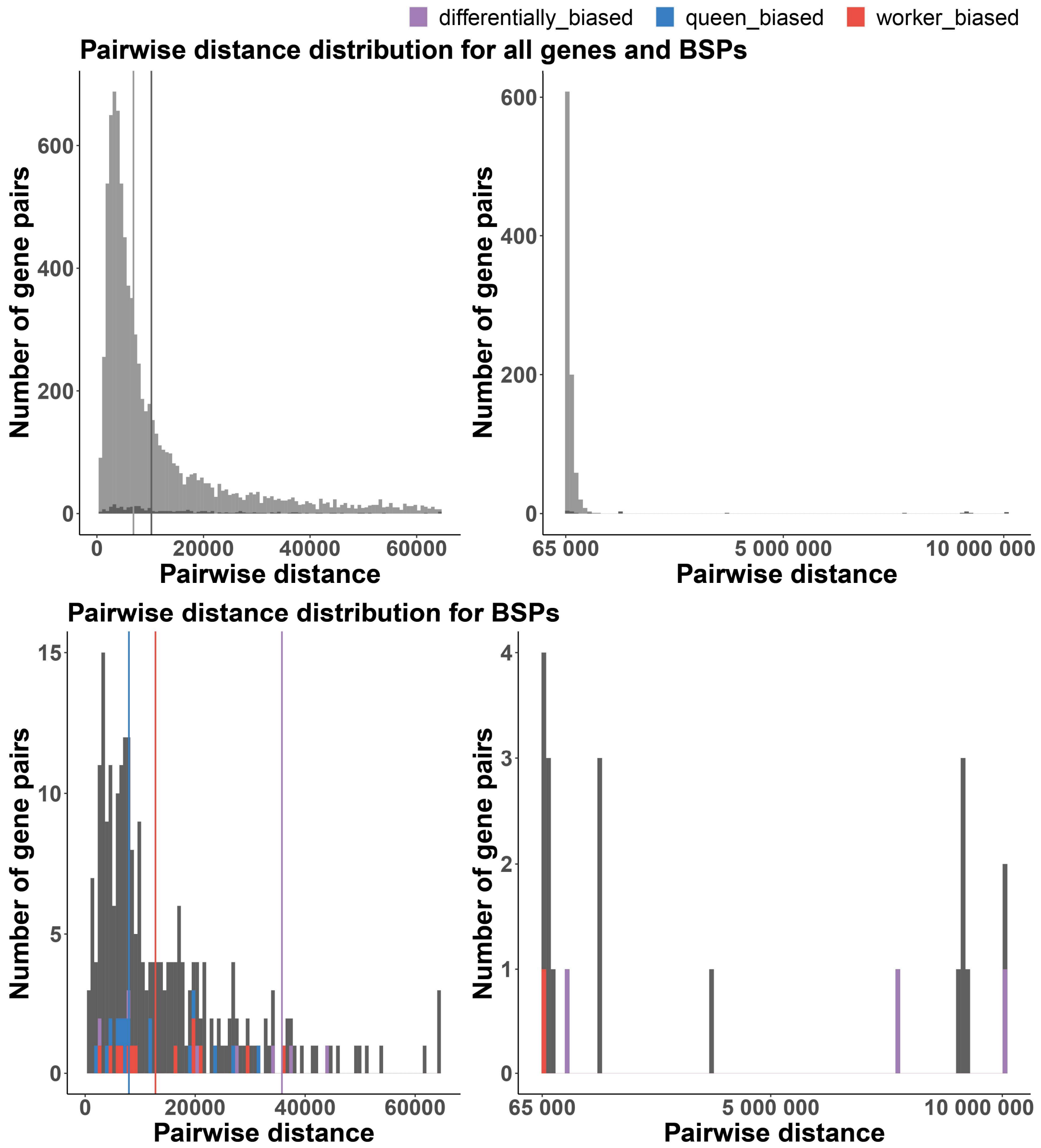

Supplementary figure S7: Pairwise distance of *Bombus*-specific paralogues and adjacent genes located in the same chromosomes. Histograms display the distribution of distance between all genes (light grey) and sets of all BSPs (dark grey) located on the same chromosome that is less than 65,000 bp from each other, and greater than 65,000 bp apart (top row). Histograms displaying the intra-chromosomal distribution of distance between all BSPs (dark grey), queen-biased BSPs (blue), worker-biased BSPs (red), and differentially-biased BSPs (purple) that are less than 65,000 bp apart and more than 65,000 bp apart, respectively (bottom row).

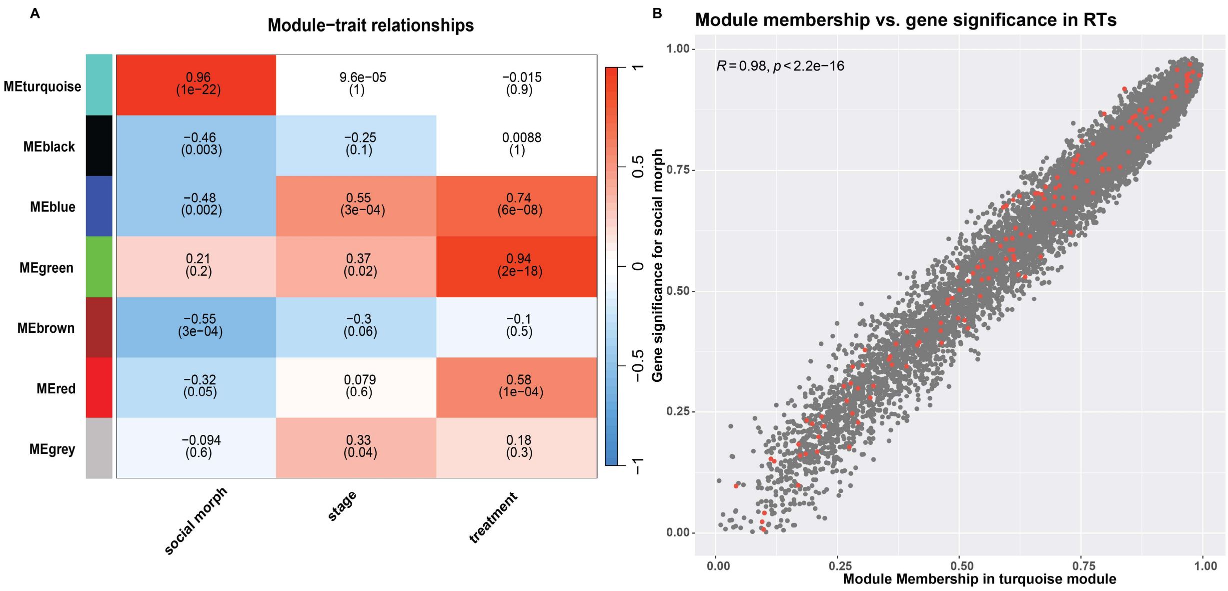

Supplementary figure S8: Weighted co-expression network analysis for genes expressed in the spermatheca and associated reproductive tissues. A) Heatmap displaying correlations between modules (gene clusters) identified by the R package WGCNA and each of social morph, stage (age of ovary development) and insemination treatment; and B) scatterplot for genes in the turquoise module displaying the correlation (Pearson's correlation coefficient) between Module Membership and gene significance for social morph in the turquoise module. Red dots represent *Bombus*-specific paralogues (BSPs).

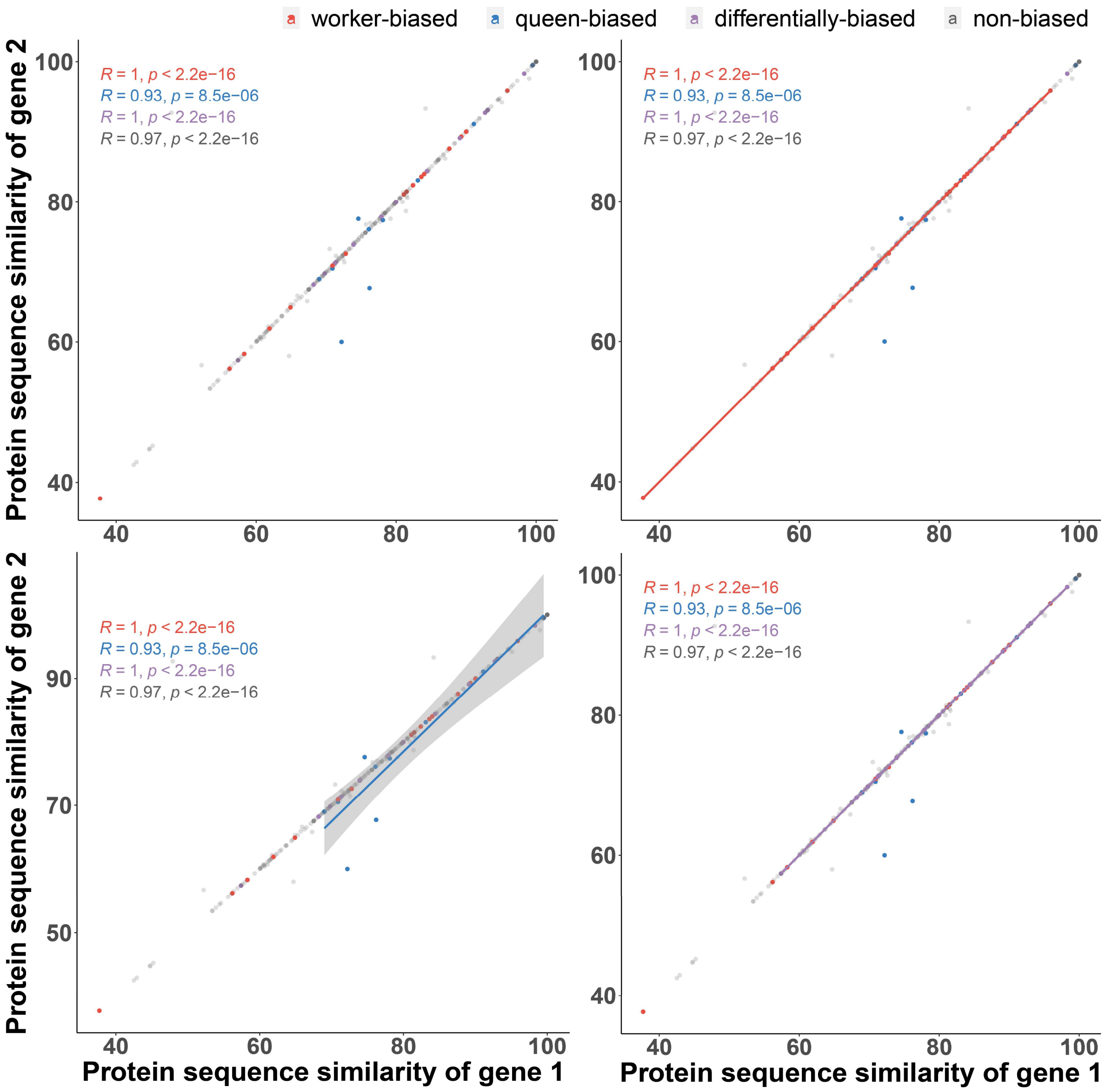

Supplementary figure S9: Correlation of protein sequence similarity between ***Bombus*****-specific paralogues.** A-D) Scatterplots displaying the correlation (Pearson's correlation coefficient) of protein sequence similarity between paralogous pairs of *Bombus*-specific paralogues (BSPs). Red dots represent worker-biased paralogues, blue dots represent queen-biased paralogues, purple dots represent differentially-biased paralogues, and grey dots represent non-biased paralogues.

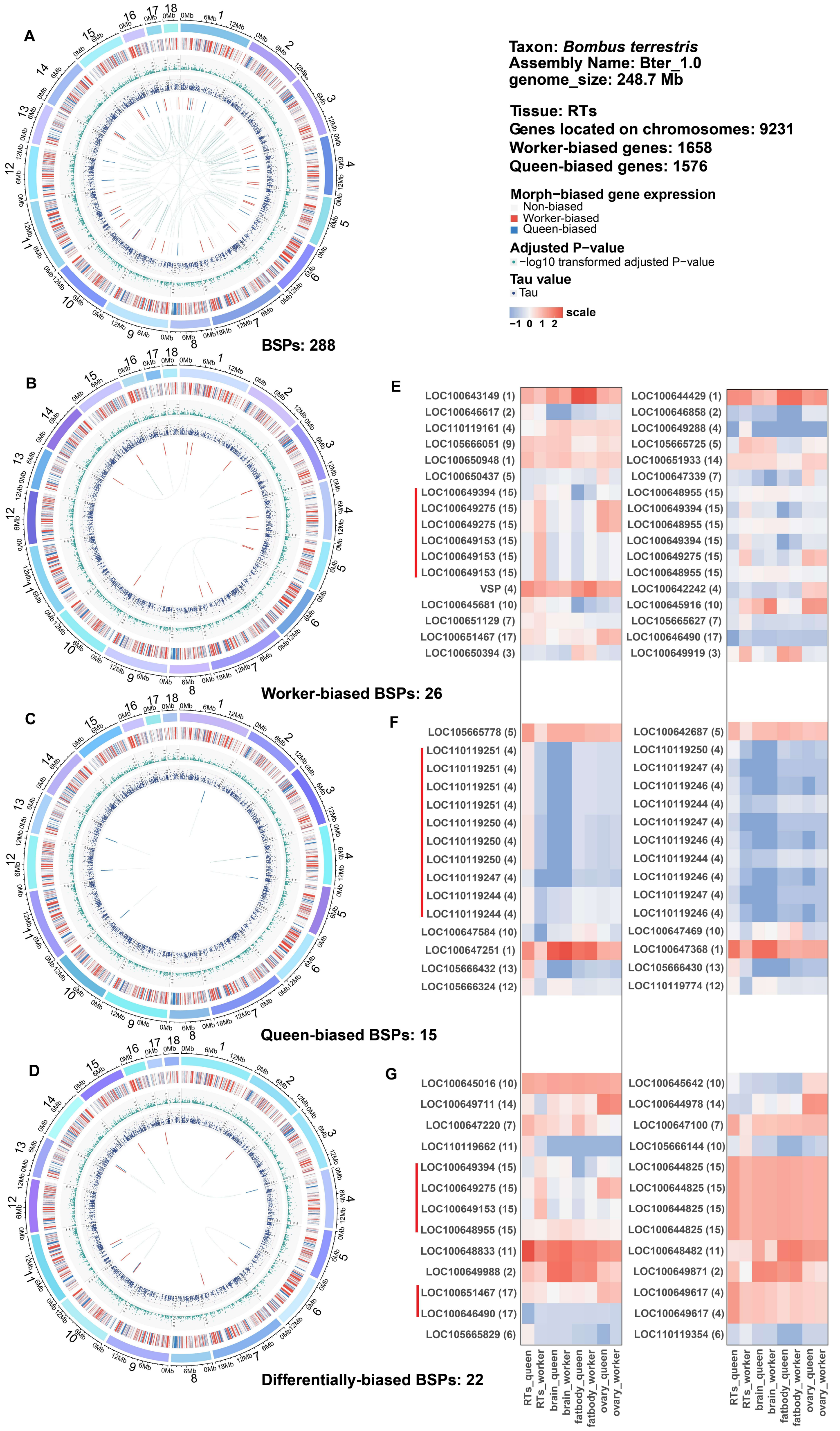
A
Taxon: *Bombus terrestris*
Assembly Name: Bter_1.0
genome_size: 248.7 Mb
Tissue: RTs
Genes located on chromosomes: 9231
Worker-biased genes: 1658
Queen-biased genes: 1576
Morph−biased gene expression
Non-biased
Worker-biased
Queen-biased
Adjusted P−value
−log10 transformed adjusted P−value
Tau value
Tau
scale
−1 0 1 2
BSPs: 288
B
Worker-biased BSPs: 26
C
Queen-biased BSPs: 15
D
Differentially-biased BSPs: 22
E
LOC100643149 (1)
LOC100646617 (2)
LOC110119161 (4)
LOC105666051 (9)
LOC100650948 (1)
LOC100650437 (5)
LOC100649394 (15)
LOC100649275 (15)
LOC100649275 (15)
LOC100649153 (15)
LOC100649153 (15)
LOC100649153 (15)
VSP (4)
LOC100645681 (10)
LOC100651129 (7)
LOC100651467 (17)
LOC100650394 (3)
LOC100644429 (1)
LOC100646858 (2)
LOC100649288 (4)
LOC105665725 (5)
LOC100651933 (14)
LOC100647339 (7)
LOC100648955 (15)
LOC100649394 (15)
LOC100648955 (15)
LOC100649394 (15)
LOC100649275 (15)
LOC100648955 (15)
LOC100642242 (4)
LOC100645916 (10)
LOC105665627 (7)
LOC100646490 (17)
LOC100649919 (3)
F
LOC105665778 (5)
LOC110119251 (4)
LOC110119251 (4)
LOC110119251 (4)
LOC110119251 (4)
LOC110119250 (4)
LOC110119250 (4)
LOC110119250 (4)
LOC110119247 (4)
LOC110119244 (4)
LOC110119244 (4)
LOC100647584 (10)
LOC100647251 (1)
LOC105666432 (13)
LOC105666324 (12)
LOC100642687 (5)
LOC110119250 (4)
LOC110119247 (4)
LOC110119246 (4)
LOC110119244 (4)
LOC110119247 (4)
LOC110119246 (4)
LOC110119244 (4)
LOC110119246 (4)
LOC110119247 (4)
LOC110119246 (4)
LOC100647469 (10)
LOC100647368 (1)
LOC105666430 (13)
LOC110119774 (12)
G
LOC100645016 (10)
LOC100649711 (14)
LOC100647220 (7)
LOC110119662 (11)
LOC100649394 (15)
LOC100649275 (15)
LOC100649153 (15)
LOC100648955 (15)
LOC100648833 (11)
LOC100649988 (2)
LOC100651467 (17)
LOC100646490 (17)
LOC105665829 (6)
LOC100645642 (10)
LOC100644978 (14)
LOC100647100 (7)
LOC105666144 (10)
LOC100644825 (15)
LOC100644825 (15)
LOC100644825 (15)
LOC100644825 (15)
LOC100648482 (11)
LOC100649871 (2)
LOC100649617 (4)
LOC100649617 (4)
LOC110119354 (6)
RTs_queen
RTs_worker
brain_queen
brain_worker
fatbody_queen
fatbody_worker
ovary_queen
ovary_worker

Supplementary figure S10: Genomic locations of BSPs and their expression patterns across bumblebee morphs and tissues (A copy of Figure 4, including more information in Circos plots). A-D) Circos plots displaying: Layer 1: Blocks representing the relative size scale of the 18 chromosomal scaffolds of the *B. terrestris* reference genome assembly (Bter_1.0); Layer 2: Each coloured, vertical band represents an individual gene, with the width of the band corresponding to the length of the gene (red bands = worker-biased genes (padj < 0.05, absolute log2FoldChange >= 1), blue bands = queen-biased genes (padj < 0.05, log2FoldChange <= -1), and grey bands = non-biased genes); Layer 3: The turquoise dots represent the values of -log10(padj) of located genes; Layer 4: The dark blue dots represent the tau values (tissue specificity) of located genes; and Layer 5: **A)** all BSPs are shown; **B)** only worker-biased BSPs are shown; **C)** only queen-biased BSPs are shown; **D)** only differentially-biased BSPs are shown. Within the centre of the circos plot, the physical genomic location of BSPs within the same paralogous category are connected by turquoise belts, with the width of the belts determined by the length of the BSPs; and **E-G)** Heatmaps displaying gene expression in each tissue and morph for BSP pairs where all genes are: (E) worker-; (F) queen-; or (G) differentially-biased BSPs, multiple pairs of genes labelled by the same red line are in the same paralogous category.

SI References

Alexa A, Rahnenfuhrer J. 2009. Gene set enrichment analysis with topGO. *Bioconductor Improv* 27:1–26.

Colgan TJ, Arce AN, Gill RJ, Ramos Rodrigues A, Kanteh A, Duncan EJ, Li L, Chittka L, Wurm Y. 2022. Genomic Signatures of Recent Adaptation in a Wild Bumblebee. *Mol. Biol. Evol.* 39:msab366.

Ferrer-Admetlla A, Liang M, Korneliussen T, Nielsen R. 2014. On Detecting Incomplete Soft or Hard Selective Sweeps Using Haplotype Structure. *Mol. Biol. Evol.* 31:1275–1291.

Kinsella RJ, Kähäri A, Haider S, Zamora J, Proctor G, Spudich G, Almeida-King J, Staines D, Derwent P, Kerhornou A, et al. 2011. Ensembl BioMarts: a hub for data retrieval across taxonomic space. *Database* 2011:bar030.

Szpiech ZA, Hernandez RD. 2014. selscan: An Efficient Multithreaded Program to Perform EHH-Based Scans for Positive Selection. *Mol. Biol. Evol.* 31:2824–2827.

Zhuang M, Colgan TJ, Guo Yulong, Zhang Zhengyi, Liu F, Xia Z, Dai X, Zhang Zhihao, Li Y, Wang L, et al. 2023. Unexpected worker mating and colony-founding in a superorganism. *Nat. Commun.* 14:5499.
